# Development of NMDAR Antagonists with Reduced Neurotoxic Side Effects: a Study on GK11

**DOI:** 10.1371/journal.pone.0081004

**Published:** 2013-11-19

**Authors:** Delphine Vandame, Lauriane Ulmann, Marisa Teigell, Monica Prieto-Cappellini, Jacques Vignon, Alain Privat, Regino Perez-Polo, Olivera Nesic, Helene Hirbec

**Affiliations:** 1 INSERM, U1051, Institut de Neurosciences de Montpellier, Montpellier, France; 2 CNRS, UMR 5203, Institut de Génomique Fonctionnelle, Labex ICST, Montpellier, France; 3 INSERM, U661, Montpellier, France; 4 Universités de Montpellier 1 & 2, UMR5203, Montpellier, France; 5 Neureva, CHU St Eloi, Montpellier, France; 6 Department of Biochemistry & Molecular Biology, UTMB, Galveston, Texas, United States of America; 7 Department of Medical Education, School of Medicine, El Paso, Texas, United States of America; Centre national de la recherche scientifique, University of Bordeaux, France

## Abstract

The NMDAR glutamate receptor subtype mediates various vital physiological neuronal functions. However, its excessive activation contributes to neuronal damage in a large variety of acute and chronic neurological disorders. NMDAR antagonists thus represent promising therapeutic tools that can counteract NMDARs’ overactivation. Channel blockers are of special interest since they are use-dependent, thus being more potent at continuously activated NMDARs, as may be the case in pathological conditions. Nevertheless, it has been established that NMDAR antagonists, such as MK801, also have unacceptable neurotoxic effects. Presently only Memantine is considered a safe NMDAR antagonist and is used clinically. It has recently been speculated that antagonists that preferentially target extrasynaptic NMDARs would be less toxic. We previously demonstrated that the phencyclidine derivative GK11 preferentially inhibits extrasynaptic NMDARs. We thus anticipated that this compound would be safer than other known NMDAR antagonists. In this study we used whole-genome profiling of the rat cingulate cortex, a brain area that is particularly sensitive to NMDAR antagonists, to compare the potential adverse effects of GK11 and MK801. Our results showed that in contrast to GK11, the transcriptional profile of MK801 is characterized by a significant upregulation of inflammatory and stress-response genes, consistent with its high neurotoxicity. In addition, behavioural and immunohistochemical analyses confirmed marked inflammatory reactions (including astrogliosis and microglial activation) in MK801-treated, but not GK11-treated rats. Interestingly, we also showed that GK11 elicited less inflammation and neuronal damage, even when compared to Memantine, which like GK11, preferentially inhibits extrasynaptic NMDAR. As a whole, our study suggests that GK11 may be a more attractive therapeutic alternative in the treatment of CNS disorders characterized by the overactivation of glutamate receptors.

## Introduction

N-Methyl-D-Aspartate receptors (NMDARs) have long been recognized as interesting therapeutic targets in many different central nervous system (CNS) disorders [1]. Overactivation of NMDARs leads to excessive influx of Ca^2+^ [2], subsequent cell death, and consequently, severe impairment of various neurological functions [3]. Thus, blocking excitotoxicity with NMDAR antagonists offers a rational approach for the therapeutic treatment of various neuropathological diseases. However, physiological activation of NMDARs is also necessary for normal brain function, so inhibition of excessive NMDAR activity must be achieved without affecting their normal physiological functions. Several potent and selective NMDAR antagonists have been developed, but their clinical approval has been prevented because of their intrinsic neurotoxicity and adverse neurobehavioural side effects [4]. Although the effectiveness of NMDAR antagonists in preventing the detrimental consequences of NMDAR overactivation has been well-documented in various neuropathological animal models [5], the failure of these molecules in clinical trials raised serious doubts as to whether sufficiently safe NMDAR antagonists can be designed [6]. 

Recent studies have shown that NMDARs play different roles depending on their subcellular localization [7]. Importantly, it was demonstrated that synaptic NMDAR activity is necessary for preserving genomic programs involved in neuronal survival [8] and is crucial for many important physiological functions [9],[10]. On the other hand, it has been shown that certain pro-death pathways are preferentially activated by extrasynaptic NMDARs [11] [12]. Therefore, it has been hypothesized that antagonists targeting extrasynaptic NMDARs would likely be safer and less harmful than NMDAR antagonists targeting synaptic receptors. 

Our group has been involved in the development of compounds based on the phencyclidine structure that led to the development of the NMDAR channel blocker GK11 [13]. Pharmacological studies have shown that GK11 binds inside the channel at a site that overlaps that of the prototypic NMDAR antagonist MK801 [14], and blocks the NMDA channels with high affinity. As a result, GK11 has potent neuroprotective properties both *in vitro* and *in vivo* [15]. Interestingly, we have reported that, in contrast to MK801, GK11 preferentially blocks extrasynaptic over synaptic NMDAR-mediated currents [16]. Moreover, preliminary dose-response studies based on qualitative histological examinations have indicated that GK11 is nearly devoid of intrinsic neurotoxicity [15].

The present study was aimed at comparing the neurotoxic profiles of GK11, MK801 and Memantine, the only NMDAR antagonist so far approved by the Federal Drug Agency (FDA). To meet this goal we have performed behavioural, histological, biochemical and transcriptomic analyses. To our knowledge, this is the only comprehensive comparison of the three most therapeutically relevant NMDAR antagonists today. We convincingly show a lower intrinsic neurotoxicity of GK11, and thus propose that this compound offers a safer therapeutic alternative to Memantine.

## Materials and Methods

### Ethics statement

Procedures involving animals and their care were conducted in strict agreement with the French Ministry of Agriculture and the European Community Council Directive no. 86/609/EEC, OJL 358, 18 December 1986. The animal studies were performed in animal facilities holding institutional licenses approved by the French Ministry of Agriculture either at the INM (N° B34-172-36) or IGF (N°D34-172-36). These studies were conducted under the supervision of Dr L. Ulmann (personal license n°34-400). Drs H. Hirbec and M. Prieto-Cappellini also hold agreements to conduct studies on animal, but their agreement numbers are pending. All necessary measures were taken to prevent animal pain.

### Drugs

GK11 [cis(pip/me)-1-[1-(2-thienyl)-2-methylcyclohexyl]piperidine] was a generous gift from Expansia (France). In the present study we used the racemic (±) compound, since this is the form that has been best characterized in terms of pharmacological and neuroprotective properties in both rodents and humans [15,17]. Additionally, (±)GK11 (hereinafter referred to as GK11) has previously been shown to be equipotent with MK801 at NMDARs [14]. All other chemicals were purchased from Sigma-Aldrich, Saint-Louis, MO, USA at the highest purity available.

### Animals and treatments

Young adult Sprague-Dawley female rats were used (220±8 g, Charles River), since they are particularly sensitive to NMDAR antagonist administration [18]. The rats were divided into groups of 12 (behaviour) or 4 to 10 animals (other studies). They were treated with a single intraperitoneal (i.p.) injection of NaCl 0.9%, GK11 (1 or 5 mg/kg), MK801 (1 or 5 mg/kg) or Memantine (20 or 50 mg/kg), respectively.

The treatment doses were determined according to the following criteria: (1) GK11 and MK801 have comparable binding affinities for NMDARs (Ki ≈ 5-10 nM,[19,20]), whereas the affinity of Memantine is 100 times lower (Ki≈700 nM, [21]); (2) GK11 and MK801 show similar neuroprotective properties in *in vitro* neuroprotection experiments ([16,22]); and (3) the doses of 1 mg/kg MK801 and GK11, and 20 mg/kg Memantine correspond to the therapeutic doses in rats [23-26]. We also decided to test a higher dose of each compound in order to explore the compounds’ safety margins. However, MK801 doses higher than 5 mg/kg could not be used for ethical reasons, as preliminary results showed that treated rats were losing more that 20% of their bodyweight and exhibiting exophthalmia. The higher doses used were thus 5 mg/kg for GK11 and MK801 and 50 mg/kg for Memantine. Additionally, initial experiments revealed that rats treated with 1 mg/kg GK11 did not show any behavioural or histological deficits, so this experimental condition was omitted in later experiments.

#### Behavioural studies

Animals were tested for general behaviour 10 min and 24h after drug administration, using an automated open-field test (Bioseb, France). Locomotive behavior was analyzed during 10 min using Acti-Track software (Bioseb, France) and the maximal and mean speeds were determined. The results are expressed as the mean ± SEM. The significance of the results was determined by a one-way non-parametric ANOVA analysis followed by Dunn’s post-tests, with p<0.05 considered significant.

#### Microarray study

Animals were divided into three groups (n=4 per group): vehicle, MK801-treated, and GK11-treated. Because our main objective was to establish whether GK11 can induce adverse drug reactions, it was administered at a dose five-fold higher than its therapeutic dose. Animals thus received a single i.p. injection of saline, 5 mg/kg GK11 or 5 mg/kg MK801. 6h after injection, animals were deeply anesthetized by i.p. injection of a lethal pentobarbital dose (Sanofi) and decapitated. The brains were removed and the posterior cingulate and retrosplenial cortices rapidly dissected out on ice, immediately placed in RNAlater^TM^ solution (Ambion) and frozen at -80°C. Animals were obtained from three independent experiments; all three experimental groups were processed in parallel.

#### qPCR validations studies

qPCR validations studies were performed on independent groups of rats that were treated as described above for the microarray studies (n=5-10 rats per experimental condition, obtained from at least two independent experiments). In these series of experiments we also included rats treated with the high dose of Memantine (50 mg/kg). Tissues from individual rats were processed simultaneously for RNA extraction, reverse-transcription and qPCR runs. 

#### Immunohistochemistry

24h or 96h after the acute injection, animals (n=3 to 6) were sacrificed and fixed by intracardiac perfusion of 4% paraformaldehyde in 0.1 M Phosphate buffer (PF 4%). Brains were dissected and post-fixed for 2h in the same solution.

### Target preparation and hybridization on Affymetrix GeneChip®

The procedures detailed in the Affymetrix GeneChip® Expression Analysis Manual (Affymetrix) were followed. Total RNA was extracted with RNAqueous kit (Ambion). The quality of the total RNA was checked using the Agilent 2100 Bioanalyzer (Agilent Technologies). Biotin-labeled cRNA was prepared using the Affymetrix RNA transcript labelling kit (Affymetrix), then fragmented. Labelled, fragmented cRNA was hybridized to a Rat Genome 230 2.0 GeneChip® microarray (Affymetrix), which was then washed, stained and scanned.

The Rat Genome 230 2.0 arrays contain > 31,000 probe sets representing > 28,000 genes. We used one microarray per rat and four rats per experimental condition. The absolute call for the presence or absence of a transcript was calculated. The proportion of probe sets designated as “present” by the Affymetrix software was 52.1±1.2%, 52.2±2.2% and 56.2±1.2% for GK11- and MK801-treated rats and controls, respectively. To increase the accuracy of the analysis, we introduced two filtering steps. First, 10,192 probes detected as “Absent” in all 12 arrays were removed. Second 17,276 ESTs (genes for which no further information exists besides the tag) were removed. After these two filtering steps, 3,631 probes were left. All further analyses were performed with this “edited’ list of probes. 

### Microarray data analysis

We essentially followed the data analysis procedure described in detail by Nesic et al. [27]. We performed: (1) cluster analysis of the overall expression profiles in order to establish similarities among arrays; (2) cluster analysis of gene expression levels in order to classify similar groups of genes; (3) identification of genes whose expression levels were significantly changed statistically (p-value<0.05) in each treated group compared to the control group; and (4) a pathway analysis to identify the relevant physiological networks. 

#### Clustering of arrays

The clustering of arrays is done in order to detect significant differences among the arrays belonging to the same experimental group. We compared the overall expression profiles of all arrays by hierarchical cluster analysis, using SPSS software®. The clustering method used Average Linkage and Manhattan distance measures between the expression vectors. 

#### Statistical significance

In order to compare the effects of MK801 and GK11 treatments, we calculated the ratio of mRNA expression values between each treated group versus the control group. We then introduced a cut-off and selected only the probes with a ratio higher than 1.5 for upregulated genes and lower than 0.66 for downregulated genes [28]. To identify the transcripts with different expression levels, we used the statistical analysis microarray software (SAM), developed at the Stanford University by Tusher et al. [29] (http://www-stat.stanford.edu/~tibs/SAM/, September 2012). At this stage, genes not detected as “present” in at least 3 out of the 4 arrays of a same experimental group were removed from the list of selected genes. We thus identified: (1) genes with statistically significant changes in expression levels (for consistently expressed genes; SAM analysis) and (2) genes that have different absolute calls (i.e. Absent/Present) between the compared groups. The changes from “Absent” to “Present” (or vice versa) indicated a consistent increase (or decrease) in mRNA levels in a treated group compared to the controls. In those cases, expression ratios were not calculated, and up- or downregulation is indicated by arrows (Table S1). 

#### Pathway analysis

Results were further analyzed using the Ingenuity Pathway Analysis software (IPA). After examination of the published literature, genes were classified according to their most relevant biological function. 

### Validation studies

#### qPCR

Total RNA from the cingulate and retrosplenial cortex was extracted as described earlier and used as a template in RT-qPCR. The quality of the total RNA was checked using the Agilent 2100 Bioanalyzer. All RNAs used in the present studies had RNA integrity numbers above 9.20. Total RNA (1 µg) was reverse transcribed using oligo dT_12-18_ primers and Superscript III (Invitrogen). The RT product was then diluted 5 times with H_2_O and stored at -20°C until use. 

Real-time PCR was performed in 96-well plates in a final volume of 10 µl using SYBR Green dye detection on the LightCycler480 system (Roche-Diagnostic). Cq for individual determination were calculated using the 2^nd^ Derivative Max tool of the LightCycler®480 software. Primers were designed with Primer 3 input software or were commercially available (Table 1). Their specificity was checked by examining the melting curve performed at the end of the qPCR run. Their efficiency was calculated by performing a standard curve by serial dilution of cDNA. The relative ratios of specifically amplified cDNAs were calculated using the ΔΔCq method [30]. The syntenin gene (NM_031986) was used as a reference gene, as its expression was found to be very stable among the 12 DNA chips (data not shown). The results are expressed as fold-changes compared to expression levels in control rats, and are shown as the mean ± SEM. Statistical analyses were performed using one-way non-parametric ANOVA analysis followed by Dunn’s post-tests, with p<0.05 considered significant.

**Table 1:** Primer sequences, amplicon size and annealing temperature for the candidate genes.

**Gene**	**Primer Sequence (5’-3’)**	**Amplicon size**	**Annealing T (°C)**	**Efficiency**	**Accession number**
Hsp 70	ACAAGGCGCAGATCCACG	121	63	2.00	NM_031971.1
	TCGTCCGGATTGATGCTCTT				
Bdnf	CCCATGGGTTACACGAAGGA	88	60	1,90	NM_012513.3
	CCCGAACATACGATTGGGTAGT				
Cyclin D1	TCTCTGGAGCCCCTGAAGAAG	133	63	1.83	X75207
	GGCGGATAGAGTTGTCAGTGTAG				
Tnfrsf4	GACCTTATAGCACTGTGAACC	113	63	2.23	NM_053552
	TGTCATGGAGTTGAGCAGGATGG				
Ilr6a	GCAGGTGGAGGTGGTGAGAAAG	189	63	1.86	NM_017020
	CTATGCTAGGACTACAGGCTGGAG				
Nos3	ATCACCAGGAAGAAGACTTTTAAGGA	102	63	2.03	AJ011116.1
	CAGGATAGTCGCCTTCACACG				
Tnfaip6	ACGATGTCCACGGCTTTGTAGG	106	63	1.73	AF159103
	ACGCATCACTCAGAAACTTCAAGG				
Tgfα	GTGGCCCTGGCTGTCCT	92	63	1,76	NM_012671.1
	CTGCAGACGAGGGCACG				
Cox2	QuantiTect Primer Assay; Qiagen		55	2.23	
	Mm_Ptgs2_1__SG,				
Stat3	GTGTCTGAATTAAGGGCAGTGAGG	104	63	1,90	NM_012747
	CCAGGGAAGGGAGAGCAATGAC				
Akt3	AAAGAAGACTGGGTTCAGAAGAGG	151	63	1,74	NM_031575
	TTGCCACTGAGAAGTTGTTGAGG				
Collagen XVIII	GTGCCCATCGTCAACCTGA	91	63	1,96	AF189709.1
	GGGCCCCAGAGTGCAGTT				
Syntenin	AGTTCTGTGTAGGTGGCAAGAGAC	78	60	1,95	NM_031986
	GGCGACAACTGAAGCACATTAGG				

#### Immunohistochemistry & quantitative analysis

Tissue blocks containing the cingulate cortex were cut into 50 µm sections using a Vibratome (Microm) and processed for immunostaining. Antigens were immunodetected using the peroxidase–antiperoxidase system [31].

The sections were examined under an Olympus Vanox microscope at a 20x magnification and analyzed blindly by an expert neuro-histologist. Images were acquired using a Nanozoomer slide scanner equipped with a 20x objective (Hammamastu). The immunopositive area (as a percent) and staining density in the posterior cingulate and retrosplenial cortex were quantified using the SAMBA image analysis system (SAMBA-Alcatel). Quantitation was performed on at least 3 rats per group. The results are expressed as the percent of immunopositive surface or the percent of density relative to control rats and are shown as the mean ± SEM. Statistical analyses were performed using one-way ANOVA analysis followed by Fisher’s LSD post-tests. 

### Cell culture

#### Cortical cell cultures & preparation of conditioned medium

High-density primary cortical cultures (250,000 cells/cm^2^) were prepared from rat brain embryos at 17 days of gestation as previously described [22]. Cultures were kept at 35 °C in a humidified atmosphere containing 5 % CO_2_ for 13 days *in vitro* (DIV), with three partial medium changes during this period. We thus obtained mature mixed neuronal and glial cell cultures.

We also prepared purified cortical astrocyte cultures from the initial cell suspension by resuspending the cells in Dulbecco's modified Eagle's medium-F12 (DMEM-F12, Life Sciences) supplemented with 10% fetal bovine serum (FBS) and 0.6% glucose. The culture medium was totally replaced 24h after plating with cold culture medium, and subsequently once a week. Using this procedure, more than 90% pure astrocyte cell cultures were obtained.

To assess the potential adverse effects of GK11 and MK801 on cortical neuron viability, 13 DIV cultures were challenged for 48h with increasing concentrations of GK11 or MK801 by replacing the culture medium with a medium devoid of serum and containing the drugs. Conditioned media were harvested after a 48h exposure to 100 µM GK11 or MK801, aliquoted and stored at -80°C. Control cultures were treated in parallel and underwent the same number of medium changes. The neuronal cultures were also fixed for immunocytochemistry and further processed for microtubule-associated protein 2 (MAP2) labelling. The MAP2 immunopositive area (as a percent), which was previously shown to be a good indicator of the neuronal viability [16], was quantified on at least 3 wells per experimental condition (10 fields analysed per well) in at least 4 series of independent experiments. The results are expressed as the mean ± SEM; statistical analyses were performed using two-way ANOVA analysis, followed by Fisher’s LSD post-tests. Conditioned media from astrocyte cultures were prepared in the same way. 

#### BV-2 cell culture

Murine microglial cells (BV-2 cell line) were obtained from PrPr E. Blasi (University of Perugia, Perugia, Italy), who originally developed this cell line [32]. They were routinely maintained in DMEM supplemented with 10% endotoxin-free FBS and 100 U/mL penicillin and 100 μg/mL streptomycin sulfate at 37 °C under 5 % CO_2_. Exponentially growing cells were plated onto 24- well plates and grown at 37 °C in a humidified atmosphere containing 5 % CO_2_. 24h after plating, the medium was replaced with the conditioned media. After overnight incubation, the BV-2 cell cultures were fixed and further processed for labeling with fluorescent conjugates of phalloidin (F-actin marker, Molecular Probes-France). The morphology of the cells was observed under a ZEISS Axiovert with a ZEISS 10X 0.3 NA EC Plan Neofluar objective. The morphology of the BV-2 cells was quantified by measuring the percentage of cells bearing at least one extension that is longer than the cell body. Quantitation was performed on at least 3 cultures per experimental condition. The results are shown as the mean ± SEM. Statistical analyses were performed using one-way ANOVA analysis followed by Bonferroni post-tests.

## Results

### Comparison of the neurotoxic effects of MK801, Memantine and GK11 (Fig. 1)

**Figure 1:**
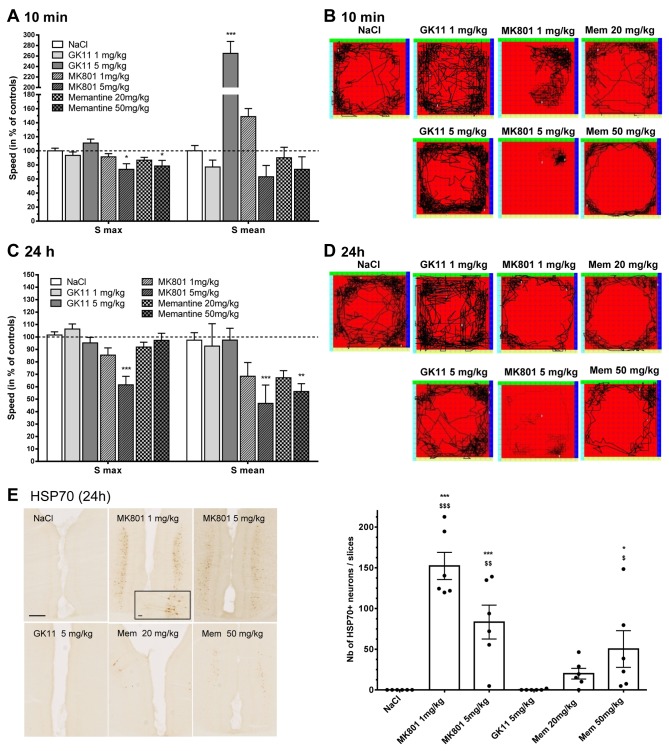
Acute and delayed effects of MK801, GK11 and Memantine treatment at the behavioural and histological levels. (**A**) Analysis of locomotive behaviour 10 min after the drug administration showed that 5 mg/kg GK11-treated rats exhibited the hyperlocomotive behaviour typically observed shortly after treatment with low dose of a high-affinity NMDAR antagonist. The same tendency was observed after 1 mg/kg MK801treatment. To the contrary, the higher doses of MK801 and Memantine (5 and 50 mg/kg, respectively) induce ataxic effects with reduced maximal speed. (**B**) Representative traces of individual rats’ routes within the arena during the observation time revealed distinct acute exploratory behaviours after the different treatments. (**C**, **D**) 24h after the treatment, GK11-treated rats behaved like control animals, whereas all other treated animals displayed ataxia and reduced exploratory behaviour. Quantitations were performed on 12 rats per group. Statistical analyses were performed using the one-way non-parametric ANOVA statistical test followed by Dunn’s post-tests. *: p<0.05; **: p<0.01; ***:p<0.001 compared to controls. (**E**) Immunohistological examination of the number of HSP70-positive neurons in the cingulate cortex. Representative images of HSP70 immunostaining: GK11 treatment did not induce any expression of HSP70, whereas a strong signal was observed in pyramidal neurons in MK801- and Memantine-treated rats (scale bar = 250 µm). Inset in MK801 1mg/kg image (scale bar 25 µm). Quantifications were performed in 6 rats per condition. Statistical analyses were performed using the one-way ANOVA statistical test followed by Fisher’s LSD post-tests. *: p<0.05; **: p<0.01; ***:p<0.001 compared to controls. $: p<0.05; $ $: p<0.01; $ $ $: p<0.001 compared to GK11-treated rats.

The NMDAR antagonist-induced alterations in normal rodent behaviour [33] have been shown to reflect their effects at the CNS level. In order to compare the potential neurotoxic effects of GK11 with those of the other well-characterized NMDAR channel blockers, MK801 and Memantine, we analyzed the effects of the three compounds on rat motor behaviour 10 min and 24h after administration. Two doses of each NMDAR antagonist were evaluated: a low dose known to be neuroprotective, and a higher dose, in order to explore the safety margin of the compounds. We thus compared the behavioural effects of 1 mg/kg and 5 mg/kg MK801 [24], corresponding doses of GK11 (1 mg/kg and 5 mg/kg, [24]); and 20 mg/kg and 50 mg/kg Memantine (whose affinity for NMDA receptors is lower than that of GK11 or MK801, [23]). We used an automated open-field test to assess the locomotor behaviour of the treated rats. We measured the maximal (S_max_) and the mean (S_mean_) velocities as the indexes of the rat locomotor activity. 

#### Acute effects of the NMDAR antagonists on motor behaviour (Figs. 1A and 1B)

As shown in Figure 1A, the highest dose of both MK801 and Memantine significantly affected the S_max_ measured for these animals. In addition, we found that S_mean_ values were significantly increased by high GK11 dose (5 mg/kg), indicating significantly increased locomotor activity elicited by the high dose of this antagonist (Figure 1A). A similar tendency was observed for the low dose of MK801; this effect failed to reach significance when all the experimental conditions were included, but when compared only to the control group, the difference was significant (p<0.001, Mann-Whitney test, not shown on the graph). Interestingly, this MK801 effect was consistent with a previous report [34]. However, the exploratory behaviours were different in GK11- and MK801-treated rats: as revealed by individual traces (Figure 1B), GK11-treated rats explored the entire space, a typical normal rat behaviour, while MK801-treated rats limited their locomotion to confined areas of the observation field. Although Memantine treatment did not significantly affect S_mean_, the exploration pattern of rats injected with the higher dose was markedly altered, as those rats typically ran parallel to the walls of the observation arena, indicating an abnormal exploratory pattern, albeit different from the ataxic exploratory behaviour of MK801-treated rats. 

#### Delayed effect of NMDAR antagonists on motor behaviour (Fig. 1C and 1D)

To investigate whether acute administration of the NMDAR antagonists could lead to more persistent behavioural changes, locomotive behaviour was tested 24h after injection. In contrast to GK11-treated rats, which exhibited locomotive behaviour similar to that of control animals whatever the dose, Figure 1C shows that at the highest tested doses MK801 and Memantine treatment induced important and significant decreases in locomotor activity. Rats treated with the higher MK801 dose were the most affected compared to controls, with significantly reduced S_max_ and S_mean_ values. At the low dose, the effect of the treatments on locomotor activity failed to reach significance when all experimental conditions were compared, but were significant when each group was compared to the control group (p<0.05, Mann Whitney test, not shown on the graph). As shown in the representative traces of rats’ routes, the exploratory aptitudes of MK801- and Memantine-treated animals were also diminished at both high and low doses, and the rats tended to run parallel to the walls of the arena. Therefore, our results suggest that GK11 has a transient stimulatory effect on locomotor activity. In contrast to MK801 and Memantine significantly decrease and persistently alter the locomotor behaviour of rats.

#### Induction of Heat Shock Protein 70 (HSP70) in the cingulate cortex (Fig. 1E)

The increased presence of HSP70-positive neurons within the cingulate cortex of rats is a useful, FDA-recommended marker of NMDAR antagonists’ neurotoxicity [35]. Therefore, we used immunohistochemistry to assess the number of HSP70-positive neurons in the cingulate cortex 24h after administration of saline, MK801, Memantine or GK11 (n=6 per group). Figure 1E shows that the delivery of even a low dose of MK801 induces strong expression of HSP70 in layer III-IV pyramidal neurons in the cingulate cortex (see inset in Figure 1E, left panel). Interestingly, we also detected the presence of HSP70-positive neurons in the same regions of 5 out of the 6 rats treated with 20 mg/kg Memantine. However, the number of HSP70-positive neurons was dose-dependent and lower than that found in MK801-treated rats (right panel). In agreement with its normal locomotive behaviour (Figure 1C and 1D), GK11 had no effect on the neuronal expression of HSP70 (Figure 1E), suggesting that this NMDAR antagonist has a markedly lower intrinsic neurotoxic potential than MK801 and Memantine.

### GK11 and MK801 have different pharmacogenomic profiles (Fig. 2)

#### DNA microarray analysis (Figs. 2A-B)

**Figure 2:**
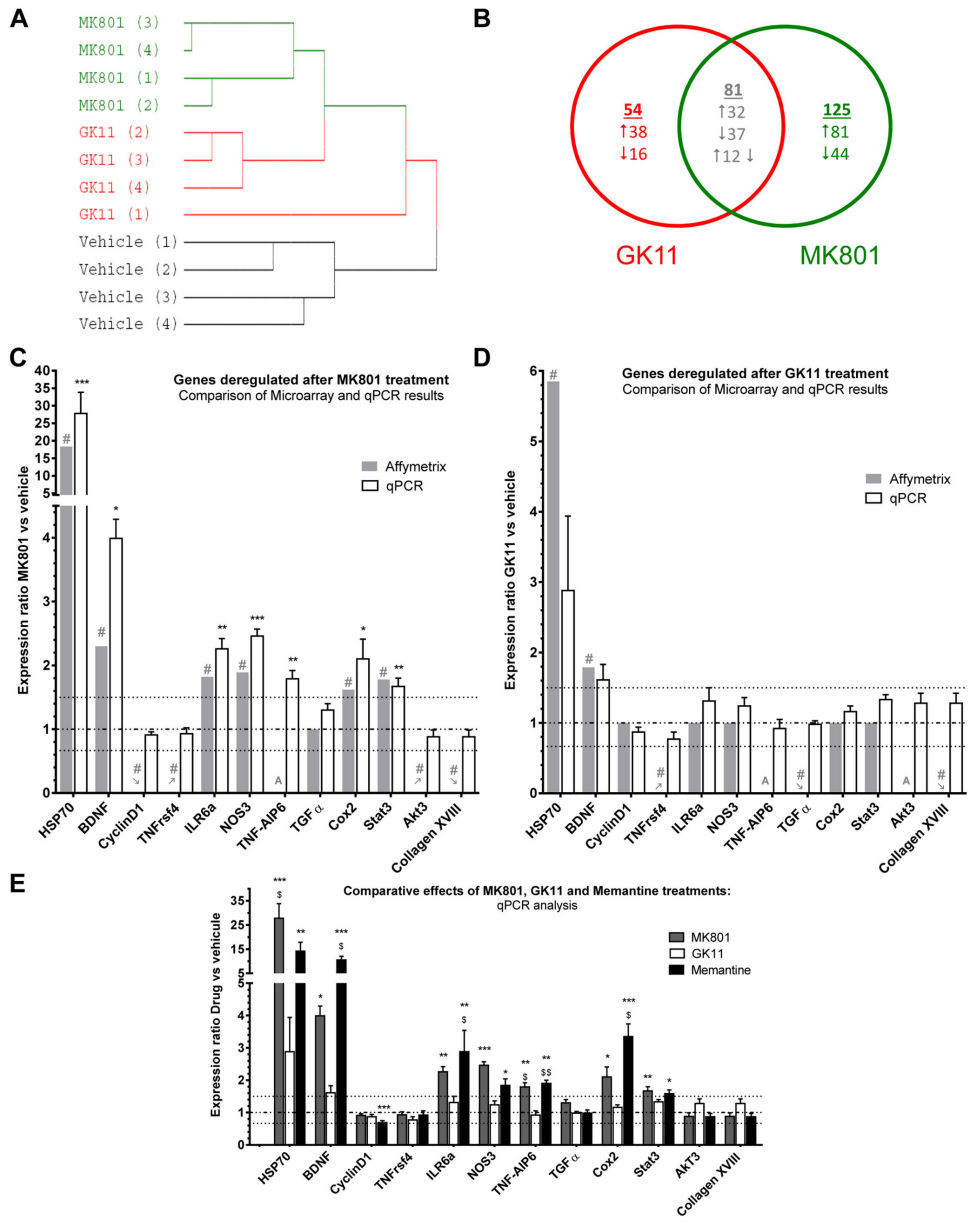
Microarray results and validation by qPCR. Comparison of the effects of MK801 and GK11 on gene expression in the cingulate cortex using microarray and qPCR analysis. (**A**) Cluster analysis of samples/arrays using average linkage between the expression levels of 3,631 probes (see methods) in the 12 Affymetrix microarrays hybridized with: Vehicle-, MK801- and GK11-treated rat cingulate cortex samples (n=4 rats/group. The farther to the right two junctions between samples are, the more dissimilar their transcription profiles. Therefore, MK801’s effect on transcription in the cingulate cortex distinctly differed from the effect of GK11, while transcription in control rats was even more dissimilar than those two treatments. (**B**) Venn diagram of genes deregulated by MK801 or GK11 treatment in the cingulate cortex. Cutoff thresholds for up- or downregulation ratios were ≥ 1.5 or ≤ 0.66, respectively. The lists of deregulated genes and the corresponding expression ratios are presented in Table S1. (**C**) Relative ratio of mRNA expression levels obtained with DNA microarrays (grey bars) and qPCR (white bars) for each candidate gene in MK801-treated rats compared to control rats. : genes induced by the treatment (i.e. absent in the control group but present in the treated group); : genes repressed by the treatment (i.e. present in the control group, but absent in the treated group; A: genes absent in all conditions. Horizontal lines represent no change (– ⋅ – ⋅ –) and cut-off fold changes of 1.5 and 0.66 (⋅⋅⋅⋅⋅⋅⋅⋅). (**D**) Relative ratio of mRNA expression levels obtained with DNA microarrays (grey bars) and qPCR (white bars) for each candidate gene in GK11-treated rats compared with control rats. (**E**) This graph summarizes the results from the qPCR validation, relative fold change of mRNA expression for each gene from 5 mg/kg MK801 (dark grey bars), 5 mg/kg GK11(white bars) and 50 mg/kg Memantine-treated rats compared to controls. **Statistical analysis**. Microarray results are presented as fold-change. # indicates significant deregulation (SAM analysis, see materials and methods). qPCR results are presented as mean ± SEM. Statistical analyses were performed using non-parametric one-way ANOVA followed by Dunn’s post-tests. *: p<0.05; ** p<0.01; ***: p<0.001 compared to controls; $: p<0.05; $ $; p<0.01; $ $ $: p<0.001 compared to GK11-treated rats.

To shed light on the molecular mechanisms underlying the decreased neurotoxic effects of GK11, we used DNA microarray analyses of transcriptional changes in the cingulate cortex measured after saline or NMDAR antagonist treatments. We thus compared the transcriptomic effects of the two NMDAR antagonists displaying the most dramatic differences in behaviour and HSP70 staining, that is 5 mg/kg MK801 and 5 mg/kg GK11. We used Affymetrix DNA microarrays with > 31,000-specific probes to analyze the transcriptional profiles of the cingulate cortex of rats treated with either GK11 (n=4), MK801 (n=4) or NaCl (n=4). Hierarchical cluster analysis of the expression levels of 3631 genes (see Materials and Methods) showed that the transcription profiles for these three experimental groups were distinctly different (Figure 2A), suggesting that a significant number of genes was differentially affected by MK801 vs. GK11. The number of genes significantly altered by GK11 or MK801 treatment is presented in a Venn diagram (Figure 2B and Table S1): MK801 and GK11 treatment affected the mRNA levels of 205 genes and 134 genes respectively, with 80 genes common to the two groups. However, the direction of mRNA expression changes and the fold- change (FC) values for those 80 genes differed depending on the treatment. For example, we found that Hsp70 mRNA expression was upregulated by 18-fold in MK801-treated rats and only by 6-fold in GK11- treated rats, consistent with MK801 eliciting a robust HSP70 protein expression (Figure 1E). 

#### qPCR validation of the microarray data (Figures 2C-E)

qPCR validation of the microarray results was performed on independent groups of animals that underwent the same type of treatments as animals used in the DNA microarray study. Of the 260 genes that were identified in microarray analysis as being affected by either or both antagonists, we examined the expression levels of 11 mRNAs (Figures 2C-E). These genes were chosen according to their deregulation profiles by the two treatments. Thus we chose genes that were deregulated after both MK801 and GK11 treatments: Hsp70, Brain-derived neurotrophic factor (Bdnf), and Collagen XVIII. We also selected genes that were deregulated by either MK801 treatement only (Interleukine 6a receptor (Ilr6a), Nitric oxide synthase 3 (Nos3), Cyclooxygenase-2 (Cox2), Signal transducer and activator of transcription 3 (Stat3), protein kinase B gamma (Akt3) and CyclinD1) or by GK11 treatment only (Transforming Growth factor α (TGFα)). In the different categories, we selected genes that were either up- or down-regulated by the treatments. Additionally, we focused on genes related to inflammation and cell survival. Of these eleven genes, microarray analysis showed that six mRNAs were significantly elevated after MK801 treatment (i.e. Hsp70, Bdnf, Ilr6a, Nos3, Cox2) and Stat3 – Figure 2C). For all these genes, qPCR experiments confirmed the increase in mRNA expression identified by microarray (Figure 2C). In GK11-treated samples, we also confirmed the results obtained with microarray analysis, with the exception of Hsp70, for which qPCR analysis did not show a statistically significant upregulation. This lack of effect may result from the large inter-individual variations in Hsp70 mRNA levels in both control and GK11-treated rats (note the large error bars in Figure 2D). However, it might also be possible that Hsp70 expression was not affected by GK11, as suggested in Figure 1E, and that the microarray results for Hsp70 changes in GK11-treated rats represent a false positive result. 

In this validation step, we also included several genes [CyclinD1, Tumor necrosis factor receptor superfamily member 4 (Tnfrsf4), Tgfα, Akt3 and Collagen XVIII] that were either induced (changed from “Absent” to “Present”) or repressed (changed from “Present” to “Absent”) by the drug treatments. However, for all these genes qPCR studies did not validate the array results (Figure 2C and 2D). 

Our qPCR results thus confirmed the data obtained with the microarray study, but only for genes whose level of expression was, under all experimental conditions, above the background level and that display consistent deregulation (i.e. 0.66>FC>1.50). In addition to the 11 genes cited above, we also included Tumor Necrosis Factor alpha-induced protein 6 (Tnfaip6) a member of the TNF family. Figure 2C and 2D show an up-regulation of this gene after MK801 but not GK11 treatment.

In the qPCR experiments we also included samples from animals treated with 50 mg/kg Memantine to compare the effects of the three treatments at the transcriptional level. Figure 2E shows that both Memantine and MK801 treatments had significant effects on the expression levels of the selected genes. On the other hand, GK11 treatment elicited markedly lower transcriptional effects.

### Pro-Inflammatory pathways are triggered by MK801, and less by GK11 (Fig. 3)

**Figure 3:**
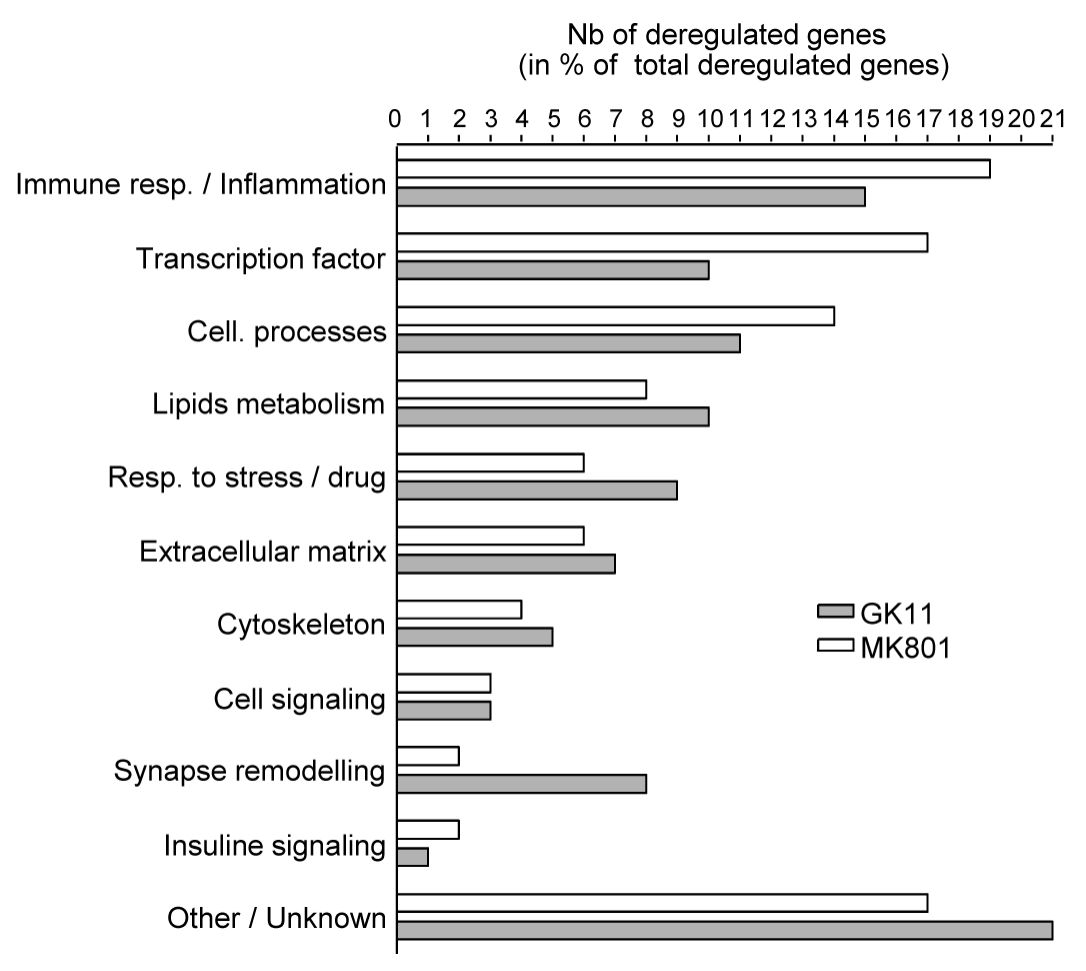
Using IPA software and analysis of the literature, the genes deregulated by either MK801 or GK11 treatment were each allocated one main biological function. Each bar represents the number of genes (expressed as the percentage of the total number of deregulated genes after each treatment) assigned to each specific biological function. Although the functions of a significant number of genes affected by the NMDAR antagonists are unknown, the most affected biofunction corresponds to the inflammatory and immune response regulators.

Ingenuity Pathways Analysis (IPA) software and analysis of the literature were used to classify the differentially expressed genes (DEGs) according to their biological functions (Table S1 and Figure 3). Figure 3 shows that after either MK801 or GK11 treatment, the DEGs had a similar distribution among the different biological functions. The lists of DEGs were also used to map the canonical pathways contained in the IPA database. Up to 29 pathways were found to be significantly altered (p-value<0.01) after MK801 treatment, but only 6 after GK11 (Table 2). Interestingly, none of the 5 pathways that were the most significantly altered in MK801-treated animals were affected by GK11.

**Table 2:** List of the most changed canonical pathways (IPA p-value <0.01).

**Ingenuity Canonical Pathways**	**p-value MK801 (n=29)**	**p-value GK11 (n=6)**	**Genes affected after MK801 treatment**	**Genes affected after GK11 treatment**
Inhibition of Angiogenesis by TSP1	3,0E-06	NS	CD47, JUN, GUCY1A3, CD36, AKT3, NOS3	JUN, CD36
Corticotropin Releasing Hormone Signaling	1,5E-05	NS	JUN, GUCY1A3, BDNF, CRH, NR4A1, PTGS2, ADCY8, NOS3, NPR2	JUN, BDNF, UCN, CRH
cAMP-mediated Signaling	2,0E-05	NS	RGS2, CREM, CAMK2D, DUSP1, GRM3, PTH1R, S1PR1, P2RY12, HTR5B, STAT3, ADCY8, PDE1C	CREM, GRM8, OPRK1, GABBR1, CHRM3
RAR Activation	3,4E-04	NS	JUN, IL3RA, DUSP1, TGFB3, AKT3, ADCY8, SMAD1, CITED2, PPARGC1A	JUN, IL3RA, SMAD1
Acute Phase Response Signaling	4,2E-04	NS	FN1, JUN, ITIH4, IL6R, AKT3, CP, STAT3, CEBPB, TNFRSF11B	JUN, C3, ITIH4, TNFRSF11B
Relaxin Signaling	4,2E-04	NS	JUN, GUCY1A3, RLN1, AKT3, ADCY8, NOS3, NPR2, PDE1C	-
Synaptic Long Term Depression	0,001	NS	GUCY1A3, GRM3, PLA2G10, CRH, LYN, ADCY8, NOS3, NPR2	GRM8, CRH, LYN
ILK Signaling	0,001	NS	FN1, JUN, PPAP2B, AKT3, ACTG2 (includes EG:72), PTGS2, CCND1, IRS3, ITGB7	-
**IL-6 Signaling**	**0,001**	**0,008**	**ABCB1, JUN, IL6R, STAT3, CEBPB, TNFRSF11B**	**ABCB1, JUN, HSPB1, TNFRSF11B**
Role of Macrophages, Fibroblasts and Endothelial Cells in Rheumatoid Arthritis	0,002	NS	FN1, JUN, WIF1, CAMK2D, LTA, CEBPD, IL6R, AKT3, STAT3, CEBPB, CCND1, TNFRSF11B	JUN, TNFRSF11B, RYK
GM-CSF Signaling	0,002	NS	CAMK2D, LYN, AKT3, STAT3, CCND1	LYN
**PI3K Signaling in B Lymphocytes**	**0,002**	**0,005**	**JUN, CAMK2D, SYK, LYN, AKT3, VAV1, IRS3**	**JUN, C3, SYK, LYN, VAV1**
HIF1α Signaling	0,003	NS	JUN, SLC2A1, AKT3, MMP24, NOS3, SLC2A3	JUN, SLC2A3
B Cell Receptor Signaling	0,003	NS	JUN, CAMK2D, SYK, EGR1, LYN, AKT3, VAV1	JUN, SYK, LYN, VAV1
G-Protein Coupled Receptor Signaling	0,003	NS	RGS2, GRM3, GHRHR, HTR5B, STAT3, CELSR2, PDE1C, HCRTR2, CAMK2D, DUSP1, PTH1R, S1PR1, P2RY12, AKT3, ADCY8	GRM8, OPRK1, GABBR1, GPR88, CHRM3, CELSR2, HCRTR2
LXR/RXR Activation	0,003	NS	APOC1, CD36, ACACA, PTGS2, TNFRSF11B	APOC1, CD36, TNFRSF11B
**LPS/IL-1 Mediated Inhibition of RXR Function**	**0,005**	**0,001**	**APOC1, ABCB1, JUN, CYP2C9, ACOX2, ACSL6, SLCO1A2, TNFRSF11B**	**APOC1, ABCB1, CYP4A14, JUN, SLC10A1, SLCO1A2, TNFRSF11B**
TR/RXR Activation	0,006	NS	SLC2A1, AKT3, ACACA, THRSP, PPARGC1A	THRB, THRSP
Glucocorticoid Receptor Signaling	0,006	NS	TSC22D3, JUN, HSPA1A, DUSP1, TGFB3, AKT3, STAT3, CEBPB, PTGS2	JUN, HSPA1A, ANXA1
**Intrinsic Prothrombin Activation Pathway**	**0,007**	**0,002**	**KLK1, THBD, COL18A1**	**KLK1, THBD, COL18A1**
Crosstalk between Dendritic Cells and Natural Killer Cells	0,007	NS	CAMK2D, IL3RA, KLRD1, LTA, ACTG2 (includes EG:72)	IL3RA, KLRD1
Fcγ Receptor-mediated Phagocytosis in Macrophages and Monocytes	0,007	NS	SYK, LYN, AKT3, VAV1, ACTG2 (includes EG:72)	SYK, LYN, VAV1
PPAR Signaling	0,008	NS	JUN, PTGS2, CITED2, TNFRSF11B, PPARGC1A	JUN, TNFRSF11B
HGF Signaling	0,010	NS	JUN, AKT3, STAT3, PTGS2, CCND1	JUN
**Disease associated pathways**				
**Huntington's Disease Signaling**	**0,001**	**0,003**	**TGM2, NSF, CAPN8, KLK1, JUN, BDNF, HSPA1A, SGK1, AKT3, GOSR2**	**TGM2, CAPN8, KLK1, JUN, BDNF, HSPA1A, GOSR2**
Colorectal Cancer Metastasis Signaling	0,004	NS	JUN, IL6R, TGFB3, AKT3, STAT3, MMP24, PTGS2, ADCY8, CCND1	-
Type II Diabetes Mellitus Signaling	0,004	NS	ACSL6, CD36, AKT3, CEBPB, IRS3, TNFRSF11B	CD36, TNFRSF11B
**Hepatic Cholestasis**	**0,009**	**0,0001**	**SLCO1C1, ABCB1, JUN, SLCO1A2, ADCY8, TNFRSF11B**	**SLCO1C1, ABCB1, JUN, SLC10A1, ABCC1, SLCO1A2, TNFRSF11B**
Hepatic Fibrosis / Hepatic Stellate Cell Activation	0,010	NS	FN1, CTGF, LEPR, IL6R, TGFB3, TNFRSF11B	LEPR, TGFA, TNFRSF11B

In agreement with the results shown in Figure 2 and Table S1, which show lower transcriptional effects after GK11 treatment, the proportion of DEGs representing transcription factors was much smaller in GK11-treated rats than in MK801-treated rats (10% in GK11-treated animals vs. 17% after MK801 treatment). Interestingly, the largest number of genes differentially affected by both antagonists belonged to the immune response and inflammatory processes (15 to 19% of the DEGs for GK11 and MK801). We thus scored the two lists of MK801 and GK11 DEGs against a custom list of 4366 genes related to Inflammation. 90 of the 206 MK801-DEGs (44%) were found on the “Inflammation” list, so the effects of MK801 treatment appeared to be strongly related to inflammatory processes (p=1x10^-5^). On the contrary, only 48 of the 135 GK11-DEGs (35%) were found to be associated with the “Inflammation” list (p=0.06).

### Differential effects of MK801, GK11 and Memantine on gliosis (Fig. 4)

**Figure 4:**
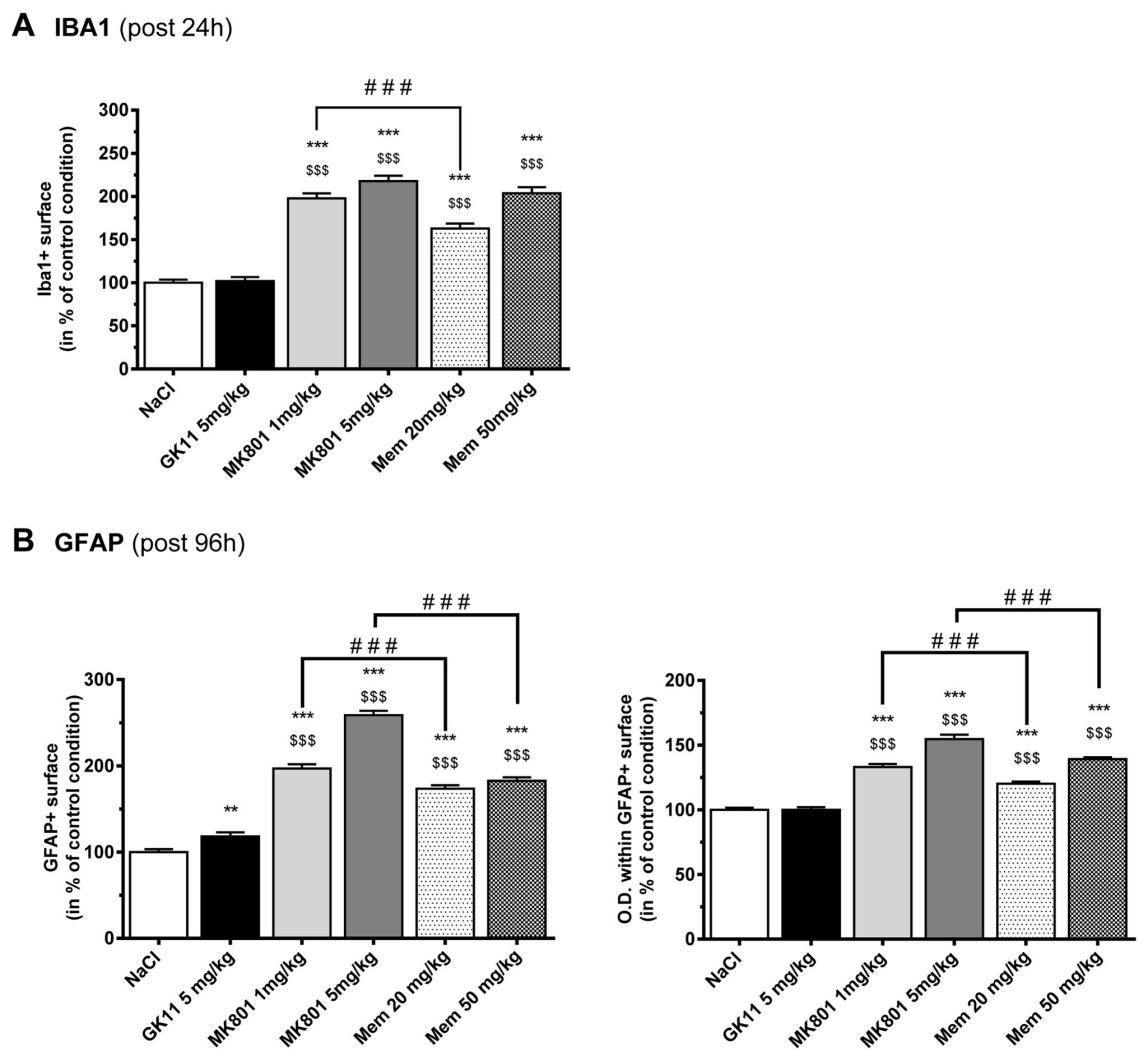
Effects of MK801, GK11 and Memantine treatment on glial and microglial activation in the rat cortex. (**A**) Quantitative analysis of the IBA1-labeled surface in the cingulate cortex 24h after the different drug treatments shows significant microglial activation after high and low doses of MK801 and Memantine treatment, but not after high-dose GK11 administration. The results are presented as the mean ± SEM (n=6 per group). (**B**) Quantitative analysis of the GFAP-labeled surface (left) and the Optical Density (right) within the labeled surface in the cingulate cortex (96h after the different drug treatments) shows significant astroglial activation after high and low doses of MK801 and Memantine treatment, but only a slight astrogliosis after high-dose GK11 administration. The results are presented as the mean ± SEM (n=3-6 per group). Statistical analyses were performed using one-way ANOVA followed by Fisher’s LSD post-tests. *: p<0.05; **: p<0.01; ***: p<0.001 compared to controls. $: p<0.05; $ $: p<0.1; $ $ $: p<0.001 compared to GK11-treated rats; #: p<0.05; ##: p<0.01; ###:p<0.001 compared to MK801-treated rats.

Activated glial cells are the hallmark of brain inflammation, so we have analyzed astrocytic and microglial activation in the cingulate cortex after MK801, Memantine or GK11 treatment. The glial fibrillary acidic protein (GFAP) and the induction of brown adipocytes-1(IBA1) are specific markers of astrocytes and microglial cells, respectively, and their expression levels can be used to quantify glial activation [36,37]. More precisely, gliosis could be characterized by either or both hyperplasia (which could be measured by an increase in the percentage of the immunopositive surface) and hypertrophy (which can lead to an increase in the percentage of the immunopositive surface, but is also characterized by an increase in the density of staining (Optical Density, or O.D.) within the immunopositive surface). Microglial activation was investigated 24h after drug treatment, whereas astrogliosis was investigated at a more delayed time (i.e. 96h), as microglial activation is thought to precede astrocytic activation [38,39]. 

As shown in Figure 4A, we found dramatic effects of both low and high doses of MK801 and Memantine on microglial activation in the cingulate cortex (i.e. IBA1 immunolabeling; n=6 rats per group). Microgliosis was mostly characterized by an increase in the immunopositive surface (+60% after the low dose of Memantine and about +100% after the high dose of Memantine or after all doses of MK801; p<0.001 vs. controls). Consistent with microscopic observations revealing the presence of amoeboid, the density of staining appeared to increase in the cingulate cortex of those animals (+ 20%, p<0.001, data not shown). In sharp contrast, no increases in the number (Figure 4A) or intensity (data not shown) of IBA1-positive cells were detected 24h after GK11 administration. 

Analysis of the GFAP staining 4 days after the single drug administration (n=3-6 rats/group) showed that both high and low doses of MK801 and Memantine elicited strong astrogliosis in the cingulate cortex. The astrocytic activation was characterized by significant increases in the GFAP-immunopositive surface (by more than 70% after Memantine and more than 100% after MK801 treatments, p< 0.001; Figure 4B left) and by increases in the density of staining (by more than 20% after Memantine and more than 30% after MK801 treatment, p< 0.001; Figure 4B right). On the other hand, GK11 treatment (5 mg/kg) only modestly increased the percentage of GFAP-immunostained surface (by 18%, p<0.01) and did not change the density of the GFAP staining of the astrocytes (100±2% of controls, p>0.05).

In summary, our results indicate that, in contrast to GK11, administration of MK801 and Memantine induces strong gliosis, further supporting a significantly lower CNS toxicity for GK11. 

### MK801-elicited glial activation is caused by neuronal injury (Figure 5)

**Figure 5:**
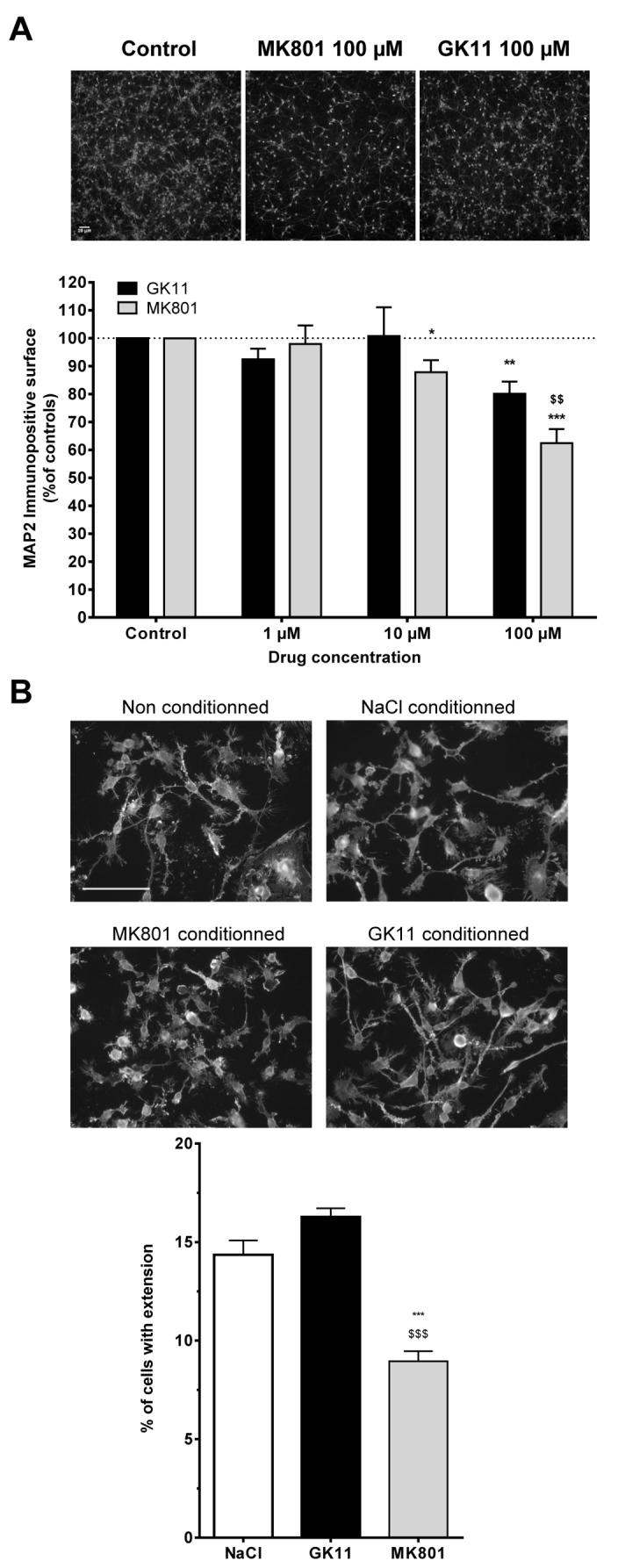
MK801-elicited microglial activation is caused by neuronal injury. (**A**) Comparison of the intrinsic neurotoxicity of GK11 or MK801 on densely seeded and mature cortical cultures. Neuronal cultures were challenged for 48h with increasing concentrations of the NMDAR antagonists. Neuronal suffering was assessed by determining the MAP2-immunopositive surface. Photographs represent typical examples of MAP2-stained neuronal cultures (Scale bar = 20 µm). Bar graph represents quantitative analysis (mean ± SEM from at least three independent experiments). Y axis: % of MAP2-positive surface in neuronal cultures treated with MK801 or GK11 normalized to the sham-treated neuronal cultures. Statistical analyses were performed using two-way ANOVA followed by Fisher’s LSD post-tests. *: p<0.05; **: p<0.01; ***: p<0.001 when compared to controls. $: p<0.05; $ $: p<0.01: $ $ $: p<0.001 when compared to GK11 treated cultures. (**B**) Effects of conditioned media obtained from Sham, MK801 and GK11-treated neuronal cultures on microglial BV-2 cells morphology. Cells treated with non-conditioned medium show a branched morphology with long processes, and only a few amoeboid cells can be seen. Cells treated with conditioned media from control (0.9% NaCl) or 100 µM GK11-treated neuronal cultures displayed similar morphology. In contrast, BV-2 cells treated with conditioned media from 100 µM MK801-treated neuronal culture show only a few short processes, and the majority of the cells had an amoeboid (“activated”) shape (Scale bar = 100 µm). The number of cells with at least one process (see Materials and Methods for further details) was quantified; the results are presented in the bar graph. The results are the mean ± SEM of quantitations performed in 3 experiments. Statistical analyses were performed using one-way ANOVA followed by Fisher’s LSD post-tests. ***:p<0.001 compared to controls; $ $ $:p<0.001 compared to GK11-treated cultures.

The effect of GK11 and MK801 on glial activation may be direct or indirect. Functional NMDARs are expressed by neurons and astrocytes [40]. A recent study also revealed a low expression of functional NMDARs on intermediate/amoeboid microglia [41], but their expression in ramified/ resting microglia has yet not been proved [42]. Therefore, the microglial cell activation observed in the present study is likely mediated via indirect mechanisms involving the inhibition of NMDA receptors in neurons and/or astrocytes. Consistent with this we found that microglial BV-2 cells directly exposed to 100 µM GK11 or MK801 were devoid of the typical morphological alterations associated with microglial activation (data not shown).

To decipher the sequence of events leading to microglial and astrocyte reactivity (Figure 5), we used a cell culture approach. We first evaluated the neuroprotective properties of the three NMDAR antagonists on mature mixed neuronal and astrocytes cortical cultures challenged by glutamate. In agreement with previous studies [20], we established that, for MK801 and GK11, complete neuroprotection was achieved for concentration of 1 to 10 µM (data not shown). On the contrary, only 70% neuroprotection could be achieved using 100 µM Memantine, and higher concentrations were toxic to the culture (data not shown). Because 100% neuroprotection could not be achieved with Memantine, this compound was not further tested.

In a second step, we evaluated the potential neurotoxic effects of the compounds on the same type of cultures by treating them with increasing concentrations of MK801 or GK11, starting with concentrations that offer complete neuroprotection against glutamate-induced toxicity.

Figure 5A shows that neurons treated for 48h with doses higher than 10 µM MK801 showed significant and dose-dependent changes in the neuritic field size, as illustrated by a significant decrease of the MAP2 immunopositive surface (10 µM: 87.8±4.3,p<0.05; 100 µM: 62.3±5.1 p <0.001; lower panel). Additionally, after MK801 treatment we observed shrunken and bright cell bodies typical of injured neurons (Figure 5A, upper panel). After 48h of GK11 treatment with 100 µM but not 10 µM, there was a small but significant reduction of the MAP2-positive surface (80.11± 4.3 with 100 µM, p< 0.01). However, whatever the dose, the GK11-treated neurons had a normal morphological appearance (Figure 5A, upper panel). Additionally, at the highest dose, the MAP2- positive surface in GK11-treated neuronal cultures was significantly greater than for the MK801-treated ones (Figure 5A lower panel, p<0.01). As a whole these results indicate that neurons exposed to MK801 *in vitro* show morphological changes resembling “injury,” while the effects of GK11 seem much less injurious. Remarkably, regardless of the drug treatment tested, astrocyte cultures appeared unaltered and without signs of activation (data not shown), suggesting that, like microglial activation, astrocytic activation in MK801-treated brains was probably indirect. Subsequent experiments with conditioned media were carried with the highest drug concentration (100 µM). 

To determine whether soluble molecule(s) secreted by neurons or astrocytes exposed to 100 μM MK801 or GK11 could trigger microglial activation, conditioned media from mixed cultures were transferred to the microglial BV-2 cell cultures and morphological changes were examined. In agreement with the literature, BV-2 cells treated with unconditioned medium exhibited thin cytoplasmic processes [43], Figure 5B). A very similar morphology was observed in cells treated with control-conditioned and GK11-conditioned media. Consistently, morphological analysis showed that the percentage of cells with at least one process was not different in control- (14.4±0.7%) vs. GK11-conditioned medium (16.3±0.9%, p>0.05). In contrast, a significant decrease in the percentage of process-bearing cells was measured after treatment with MK801-conditioned medium (8.9±1.0%; p<0.001 compared to control or GK11), with the cells displaying an amoeboid form typical of activated microglia. Importantly, exposure of BV-2 cultures to treated astrocyte- conditioned media did not affect microglial activation (data not shown), ruling out a potential role of astrocytes in triggering the observed microglial activation. 

Our results therefore suggest that MK801 provokes much more harmful neuronal changes than does GK11. Neuronal injury caused by MK801 could elicit the release of factors that in turn activate microglia and trigger the inflammatory reactions indicated in our microarray analyses. Activation of microglial cells would then likely perpetuate the neurotoxic effects of MK801 via direct action at neurons, or indirectly by activating astrocytes. In contrast, GK11 exhibited a markedly lower pro-inflammatory potential and resultant neurotoxicity. 

## Discussion

The main goal of this study was to compare the potential adverse effects of GK11 with those of MK801, the prototypic NMDA channel blocker. In selected experiments we also included Memantine, the only NMDAR antagonist currently approved for the treatment of moderate to severe forms of Alzheimer’s disease [23]. 

Activation of NMDARs is essential for brain physiology; however, overactivation of these receptors is also the primary cause of neuronal cell death in many CNS pathologies [44]. These opposite effects of NMDAR activation on cell fate may arise from differentially located receptors, e.g. at synaptic vs. extrasynaptic locations [45]. For example, calcium signaling through synaptic NMDARs induces a coordinated upregulation of pro-survival and downregulation of pro-death genes (i.e., a “survival program”, [8]). In contrast, activation of extrasynaptic NMDARs fails to activate this neuroprotective program, but induces the expression of pro-cell-death genes [8]. In addition, it has been shown that synaptic and extrasynaptic NMDARs have opposite effects on CREB (cAMP response element binding protein) functions or signaling molecules such as extracellular signal-regulated kinases 1 and 2 (ERK1/2; [46,47]).

It is well established that MK801 is an efficient blocker of both synaptic and extrasynaptic NMDARs [48], whereas we have shown that GK11 is a more effective inhibitor of extrasynaptic receptors [16]. Memantine has also been shown to preferentially antagonize extrasynaptic NMDARs [48,49], and was thus included in our study. Parallel comparison of the acute and delayed effects of GK11, MK801 and Memantine administration showed that at moderately high doses, MK801 and Memantine elicit abnormal and worrisome perturbations of locomotive behaviors in rats: both locomotor activity and exploratory capacity were altered. Our results also show that lasting alterations of the locomotive activity and exploratory behaviour could be measured for the neuroprotective doses of MK801 and Memantine; however, these changes were weaker than for the higher doses. Importantly, the behavioural alterations were associated with neurotoxic effects at the level of the cingulate cortex. In contrast, even high doses of GK11 provoked only transient locomotor alterations without evidence of neurotoxic effects. Neuroprotective doses of GK11 had no acute or delayed behavioural or histological effects.

To elucidate the molecular changes underlying the differences between GK11 and MK801, we used a genome-wide approach to compare their transcriptional signatures. We found very different profiles for the two drugs. Changes in gene expressions after treatment with different NMDAR antagonists have already been reported [27,50-55]. Our findings are consistent with those previous studies, as the genes identified are either the same (i.e. Hsp70, Bdnf, Stat3, Crh, Crem/Icer) or with similar biological functions (i.e. inflammation-related genes, [27]). However, those earlier studies were based on single-gene or low-throughput approaches (at most 7,000 genes [55]). Thus this study is, to our knowledge, the first to investigate the transcriptional effects of NMDAR antagonists at the level of the entire rat genome. 

We found that DEGs affected by either or both GK11 and MK801 were associated with the same Gene Ontology families; however, there was only a moderate overlap (31%) between the identified genes. Interestingly, out of the 29 canonical pathways affected by MK801 administration, GK11 affected only 6, while no canonical pathway was specific to GK11. Most notably MK801 affected a large number of genes involved in inflammation regulation, whereas the effects of GK11 were much weaker. Among the 5 top pathways specifically affected by MK801 treatment but not by GK11, we found Corticotropin Releasing Hormone and Acute-phase Response signalling and RAR activation, all recognized as playing important roles in response to stressful conditions (see IPA software, http:// http://www.ingenuity.com, for the description of these biofunctions). Biofunctions associated with altered gene expressions after the two treatments were also differentially affected: the apoptosis- related “Cell Death/Compromise” biofunction was more altered in MK801 samples (99 DEGs compared to 51 in GK11 samples). Additionally, the main biofunction affected after GK11 treatment is “Lipid Metabolism” (39 genes), but with 48 DEGs associated with this specific biofunction, MK801 has a greater impact on it. In summary, our microarray study demonstrated that MK801 administration strongly affected genes involved in cell degeneration and in the immune/inflammatory responses, which explains significant changes in HSP70, an accepted marker of neuronal stress [35]. In agreement with previous studies [50,52] we found that MK801 treatment induces a strong upregulation of Hsp70 mRNA, which was confirmed by qPCR. Furthermore, our immunohistological analyses showed that Hsp70 mRNA changes were translated to the HSP70 protein level. The importance of our validation studies (i.e. qPCR and/or immunohistochemistry) in eliminating false positive results, not uncommon in microarray analyses [29], was evident in the analysis of GK11’s effect on the gene encoding HSP70. Although the microarray indicated that GK11 treatment increased Hsp70 mRNA expression by 6-fold, qPCR assays failed to detect any significant upregulation of this mRNA, and the histological analysis showed no HSP70 -labelled neurons in the cortex of GK11-treated rats. In the group of inflammatory regulators identified by microarray analyses selectively induced by MK801, but not GK11, qPCR confirmed the up-regulation of several key markers and/or regulators of inflammation, e.g. Cox2, Il6ra, TNFα and Nos3 [56,57], thus further validating our conclusion that MK801 affects a plethora of inflammatory processes, in contrast to GK11. Interestingly, the qPCR experiments also show that Memantine treatment induces the upregulation of the same set of genes as MK801. The fold-changes induced by the two drugs were of similar magnitude, thus indicating that Memantine also induces strong transcriptional effects.

To further elucidate the cellular basis of inflammatory reactions induced by MK801, but not by GK11, we analyzed their effects, and those of Memantine, on the time course and extent of gliosis in the cingulate cortex. In agreement with previous studies [18,58,59], MK801 induced lasting and marked gliosis that included both astrogliosis and microglial activation. This persistent deleterious effect is consistent with (1) the deregulation of numerous transcription factors evidenced by our microarray analyses and (2) the severe disturbance of synaptic plasticity, and the associated lasting memory impairments observed in rats [33]. In addition, our *in vitro* results also suggest for the first time that MK801 activates microglia indirectly, likely via the release of factors by injured/stressed neurons. The nature and identity of these NMDAR-dependent neuronal factors capable of inducing microglial activation remains to be determined. Additionally, the mechanisms involved in NMDAR antagonist-mediated neuronal injury still needs to be further elucidated; however, one can speculate that MK801, by blocking synaptic NMDARs, turns off the normally active “survival program” driven by these receptors [8], leading to neuronal degeneration and the release of danger (or the retention of survival) signalling molecules. Changes in the expression level of these molecules might then induce microglial and astroglial activation. Activation of microglia and/or astrocytes is unlikely to be the first step towards neuronal degeneration, as MK801 did not seem to affect these glial cells even at high concentrations. However, microglial and astrocytic activation could lead to the release of pro-inflammatory molecules and thus play a direct role in the propagation of the neurodegenerative process. Conversely, the lack of gliosis in GK11-treated rats strongly supports a safer profile for this drug. 

One of the strengths of the present study is that it allows the direct comparison of the behavioural and inflammatory effects of GK11 with those of the only clinically approved NMDAR channel blocker, Memantine. Memantine’s adverse effects, such as psychotomimetic effects in humans [60] or impairment of memory in rats [61] and the expression of HSP70 in the limbic rat cortex [53,62] have already been described. However, to our knowledge, this study is the first to demonstrate that Memantine effects are long-lasting, with persistent astrogliosis even 4 days after a single administration. Taken together our data indicate that neuroprotective Memantine doses also produce deleterious side effects. Reported Memantine beneficial effects (currently investigated in the Clinical trials involving Alzheimer patients; [63]) may be associated with interactions with other transmitter systems such as cholinergic receptors [64]. However, the potentially neurotoxic effects of Memantine should be investigated further and taken into account as possible adverse side effects in treated patients. 

In summary, this study shows that neuronal injury and microglial /astroglial activation are high in MK801-, moderate in Memantine- and low in GK11-treated animals. The difference between the extrasynaptic and/or synaptic effects of MK801, GK11 or Memantine may explain the prominent and well-established toxicity of MK801 vs. GK11/Memantine, but cannot fully explain the results of our study showing a greater safety of GK11 over Memantine. One explanation for the lower neurotoxicity observed with GK11 is that this compound is only a partial blocker of the NR2A- containing NMDARs [16], while Memantine blocks NR2A- and NR2B-containing receptors with the same potency [65], suggesting that the design of a safe NMDAR antagonist should take into account not only the subcellular site of the antagonist’s action (synaptic vs. extrasynaptic), but also the subunit composition of the NMDARs (NR2A vs. NR2B) that will be blocked by the antagonist. Given that these two parameters may not be independent (NR2B-containing receptors account for 70% of the extrasynaptic NMDA currents and the NR2A-containing receptors for 70% of the synaptic NMDA currents [66]), further elucidation of the contribution of the NMDAR subtypes to the safety of GK11 is warranted. Interestingly, several recent studies on NMDARs’ allosteric modulators point towards an improved therapeutic index with the NR2B-subtype-selective approach (for review 67).

We have previously shown that GK11 also interacts with “non-NMDA” binding sites [68]. The molecular nature of these binding sites is currently unknown, and we cannot rule out that this interaction could contribute to the lower intrinsic neurotoxicity of this compound. However, “non-NMDA” binding sites are unlikely to represent major contributors to GK11’s low neurotoxicity, as this compound has only a low affinity for those sites (about 100 nM). Similarly, previous pharmacological studies have shown that among more than 40 neurotransmitter receptors tested, GK11 only weakly interacts with muscarinic (in the sub-micromolar range) and dopaminergic (in the micromolar range) receptors [68]. Therefore, interaction of GK11 with other CNS receptors is unlikely to explain the low intrinsic neurotoxicity of this compound.

The side-by-side comparison of the three NMDAR channel blockers in our study revealed that even at high doses GK11 (1) had only transient effects on rat locomotor behaviour (2), did not elicit sign of neuronal injury (3), did not induce detectable microglial activation, and (4) elicited only minor astrogliosis, in contrast to the other two NMDAR antagonists. Therefore, our study seems to demonstrate a significantly safer profile of GK11 over Memantine. Our findings are in agreement with results from Phase-I and Phase‑IIb clinical trials for spinal cord injury and head trauma [69], which showed no deleterious side effects of GK11 in more than 200 treated patients. 

In conclusion, our report suggests that GK11 could be an attractive clinical drug targeting NMDARs, and that would merit consideration for the treatment of numerous CNS diseases associated with glutamate excitotoxicity. Compared to other NR2B-selective antagonist such as Ifenprodil, which binds to the extracellular lobes of the receptors [67], GK11 offers the advantage of being use-dependent, so it would preferentially block NMDARs that are continuously or repetitively activated, as may be the case in pathological conditions, while leaving those that are physiologically activated relatively unaffected. Moreover, as a more lipophilic molecule, a lower dose of the compound may be required for systemic administration. 

## Supporting Information

Table S1 **List of genes deregulated after MK801 and/or GK11 treatment.** NB: Grey-colored lines correspond to genes used in qPCR validation studies.
(PDF)
