# Supplementary material for: Development of NMDAR Antagonists with Reduced Neurotoxic Side Effects: a Study on GK11
Source: PLoS One. 2013 Nov 19;8(11):e81004. doi: 10.1371/journal.pone.0081004 (PMC3834252; doi:10.1371/journal.pone.0081004)
Supplement: Table S1 — List of genes deregulated after MK801 and/or GK11 treatment. NB: Grey-colored lines correspond to genes used in qPCR validation studies. (PDF) [file pone.0081004.s001.pdf]

Family	Symbol	Entrez Gene Name	Affymetrix	Fold Chge GK11	Fold Chge MK801	Type(s) IPA classification	Location
**Genes deregulated after both MK801 and GK11 treatments**							
Cellular processes -Death	CAPN8	calpain 8	1387991_at	1,66	↘	endopeptidase	Cytoplasm
Cellular processes - Proliferation	CSH1	chorionic somatomammotropin hormone 1	1367587_at	1,45	↘	growth factor	Extracellular Space
Cellular processes -Death	BOK	BCL2-related ovarian killer	1369084_a_at	1,88	↘	other	unknown
Cellular processes -Death	SLC2A3	solute carrier family 2 (facilitated glucose transporter), membe	1387707_at	↘	2,33	transporter	Plasma Membrane
Cellular processes -Growth	RET	ret proto-oncogene	1370989_at	1,76	1,96	kinase	Plasma Membrane
Cellular processes -Death	HOMER1	homer homolog 1 (Drosophila)	1370454_at	2,01	2,04	Scafolding protein	Plasma Membrane
Cellular processes -Growth	EPHA5	EPH receptor A5	1371007_at	-1,42	-1,86	kinase	Plasma Membrane
Cellular processes -Growth	TPM1	tropomyosin 1 (alpha)	1370513_at	-1,65	-1,57	other	Cytoplasm
Cellular processes -Death	HK1	hexokinase 1	_Rat_Hexokinas	↘	-1,70	kinase	Cytoplasm
Cellular processes -Death	AMBRA1	autophagy/beclin-1 regulator 1	1369601_at	↘	↘	other	unknown
Cellular processes -Growth	CA3	carbonic anhydrase III, muscle specific	1367896_at	↘	-1,95	enzyme	Cytoplasm
Cellular processes - Death	TGM2	transglutaminase 2	1369943_at	↗	↗	enzyme	Cytoplasm
Cytoskeleton	CELSR2	cadherin, EGF LAG seven-pass G-type receptor 2 (flamingo h	1371018_at	1,49	1,50	G-protein coupled receptor	Plasma Membrane
Cytoskeleton	NEXN	nexilin (F actin binding protein)	1370697_a_at	↘	↘	other	Plasma Membrane
Cytoskeleton	TMOD1	tropomodulin 1	1387370_at	↘	↘	enzyme	Cytoplasm
Extracellular matrix	DSPP	dentin sialophosphoprotein	1368743_a_at	↘	1,92	other	Extracellular Space
Extracellular matrix	LOX	lysyl oxidase	1368171_at	2,33	2,01	enzyme	Extracellular Space
Extracellular matrix	FXYD5	FXYD domain containing ion transport regulator 5	1368207_at	2,08	2,20	ion channel	Plasma Membrane
Extracellular matrix	COL5A1	collagen, type V, alpha 1	1369955_at	↘	↘	other	Extracellular Space
Extracellular matrix	COL18A1	collagen, type XVIII, alpha 1	1388143_at	↘	↘	other	Extracellular Space
Extracellular matrix	IBSP	integrin-binding sialoprotein	1368416_at	↗	↗	other	Extracellular Space
Response to drug / stress	ABCB1	ATP-binding cassette, sub-family B (MDR/TAP),mb1	1370464_at	-1,98	-2,09	transporter	Plasma Membrane
Response to drug / stress	HSPA1B	heat shock 70kDa protein 1B	1368247_at	**5,85**	**18,35**	stress markers	Cytoplasm
Response to drug / stress	HSPA1A	heat shock 70kDa protein 1A	1370912_at	**3,68**	**5,09**	stress markers	Cytoplasm
Response to stress / drug	ITIH4	inter-alpha (globulin) inhibitor H4	1368707_at	↗	↗	other	Extracellular Space
Response to stress / drug	RGD:708417	UDP glucuronosyltransferase 2 family, polypeptide B7	1370615_at	↗	↗	enzyme	Cytoplasm
Immune response / Inflammation	GBP2	guanylate binding protein 2, interferon-inducible	1368332_at	1,52	↘	enzyme	Cytoplasm
Immune response / Inflammation	TRIB1	tribbles homolog 1 (Drosophila)	1371019_at	↘	1,63	kinase	Cytoplasm
Immune response / Inflammation	BDNF	brain-derived neurotrophic factor	1368677_at	→	**2,30**	growth factor	Extracellular Space
Immune response / Inflammation	BDNF	brain-derived neurotrophic factor	1368678_at	**1,79**	→	growth factor	Extracellular Space
Immune response / Inflammation	THBD	thrombomodulin	1368901_at	2,00	2,53	transmembrane receptor	Plasma Membrane
Immune response / Inflammation	CRH	corticotropin releasing hormone	1369303_at	2,15	2,28	stress markers	Extracellular Space
Immune response / Inflammation	SLCO1A2	solute carrier organic anion transporter family, member 1A2	1387093_at	-1,94	-2,09	transporter	Plasma Membrane
Immune response / Inflammation	TNFRSF11B	tumor necrosis factor receptor superfamily, member 11b	1369407_at	-1,71	-2,24	transmembrane receptor	Plasma Membrane
Immune response / Inflammation	SLC15A3	solute carrier family 15, member 3	1370516_at	↘	↘	transporter	unknown
Immune response / Inflammation	IL3RA	interleukin 3 receptor, alpha (low affinity)	1387935_at	↘	↘	transmembrane receptor	Plasma Membrane

Family	Symbol	Entrez Gene Name	Affymetrix	Fold Chge GK11	Fold Chge MK801	Type(s) IPA classification	Location
Immune response / Inflammation	SYK	spleen tyrosine kinase	1368186_a_at	↗	↗	kinase	Cytoplasm
Immune response / Inflammation	LYN	v-yes-1 Yamaguchi sarcoma viral related oncogene homolog	1368679_a_at	↗	↗	kinase	Cytoplasm
Immune response / Inflammation	KLRD1	killer cell lectin-like receptor subfamily D, member 1	1369306_at	↗	↗	transmembrane receptor	Plasma Membrane
Immune response / Inflammation	IL9R	interleukin 9 receptor	1369359_at	↗	↗	transmembrane receptor	Plasma Membrane
Immune response / Inflammation	TNFSF4	tumor necrosis factor (ligand) superfamily, mb4 4	1369481_at	↗	↗	cytokine	Extracellular Space
Immune response / Inflammation	AZGP1	alpha-2-glycoprotein 1, zinc-binding	1387234_at	↗	↗	transporter	Extracellular Space
Insulin signalling	PCSK1	proprotein convertase subtilisin/kexin type 1	1387247_at	1,66	1,78	peptidase	Extracellular Space
Insulin signalling	SCNN1G	sodium channel, nonvoltage-gated 1, gamma	1387986_at	↘	↘	ion channel	Plasma Membrane
Lipid metabolism	CD36	CD36 molecule (thrombospondin receptor)	1367689_a_at	2,62	↘	transmembrane receptor	Plasma Membrane
Lipid metabolism	CBR1	carbonyl reductase 1	1368037_at	1,60	1,61	enzyme	Cytoplasm
Lipid metabolism	PLA1A	phospholipase A1 member A	1370445_at	1,94	2,10	enzyme	Extracellular Space
Lipid metabolism	VLDLR	very low density lipoprotein receptor	1387455_a_at	-1,53	-1,64	transporter	Plasma Membrane
Lipid metabolism	APOC1	apolipoprotein C-I	1368587_at	↘	↘	transporter	Extracellular Space
Lipid metabolism	ACADSB	acyl-Coenzyme A dehydrogenase, short/branched chain	1369526_at	↘	↘	enzyme	Cytoplasm
Lipid metabolism	EBP	emopamil binding protein (sterol isomerase)	1386990_at	↘	↘	enzyme	Cytoplasm
Lipid metabolism	THRSP	thyroid hormone responsive (SPOT14 homolog, rat)	1387852_at	↗	↗	other	Nucleus
Lipid metabolism	LEPR	leptin receptor	1388260_a_at	↗	↗	transmembrane receptor	Plasma Membrane
Transcription factors	VAV1	vav 1 guanine nucleotide exchange factor	1369387_at	1,82	↘	transcription regulator	Nucleus
Transcription factors	JUN	jun oncogene	1369788_s_at	1,48	1,71	transcription regulator	Nucleus
Transcription factors	SMAD1	SMAD family member 1	1369174_at	↘	-2,50	transcription regulator	Nucleus
Transcription factors	N5	DNA binding protein N5	1369438_at	↘	-2,19	transcription regulator	Nucleus
Transcription factors	NR1D1	nuclear receptor subfamily 1, group D, member 1	1370816_at	-1,64	-1,76	ligand-dependent nuclear rec	Nucleus
Transcription factors	KHDRBS1	KH domain containing, RNA binding, signal transduction asso	1386896_at	↘	↘	transcription regulator	Nucleus
Transcription factors	MTPN	myotrophin	1387786_at	-1,42	-1,71	transcription regulator	Nucleus
Transcription factors	BMYC	brain expressed myelocytomatosis oncogene	1388274_at	↘	↘	other	Nucleus
Transcription factors	UNCX4.1	UNC homeobox	1369413_at	↗	↗	transcription regulator	unknown
Transcription factors	CREM	cAMP responsive element modulator	1369737_at	↗	↗	transcription regulator	Nucleus
Cell Signaling	HCRTR2	hypocretin (orexin) receptor 2	1369377_at	↘	↘	G-protein coupled receptor	Plasma Membrane
Synapse formation	DRP2	dystrophin related protein 2	1371224_a_at	↘	↘	other	Plasma Membrane
Others / unknown	CLCN4	chloride channel 4	1369791_at	1,95	↘	ion channel	Plasma Membrane
Others / unknown	DCTPP1	dCTP pyrophosphatase 1	1370308_at	↘	1,80	enzyme	unknown
Others / unknown	SCN7A	sodium channel, voltage-gated, type VII, alpha	1370973_at	↘	2,07	ion channel	Plasma Membrane
Others / unknown	LOC683655	similar to ADAM 22 precursor (A disintegrin and metalloprotei	1385854_at	1,67	2,04	endo-peptidase	unknown
Others / unknown	NLN	neurolysin (metallopeptidase M3 family)	1369669_at	-2,13	-1,91	metallo-peptidase	Cytoplasm
Others / unknown	IPMK	inositol polyphosphate multikinase	1368340_at	-1,49	-1,62	IP kinase	Nucleus
Others / unknown	SYNJ1	synaptojanin 1	1370070_at	-1,70	-1,88	phosphatase	Cytoplasm
Others / unknown	SLCO1C1	solute carrier organic anion transporter family, member 1C1	1370205_at	-1,58	-1,63	transporter	Plasma Membrane

Family	Symbol	Entrez Gene Name	Affymetrix	Fold Chge GK11	Fold Chge MK801	Type(s) IPA classification	Location
Others / unknown	CACNA1H	calcium channel, voltage-dependent, T type, alpha 1H subuni	1368398_at	↘	↘	ion channel	Plasma Membrane
Others / unknown	CLCNKB	chloride channel Kb	1368765_at	↘	↘	ion channel	Plasma Membrane
Others / unknown	SLC38A4	solute carrier family 38, member 4	1369074_at	↘	↘	transporter	unknown
Others / unknown	GOSR2	golgi SNAP receptor complex member 2	1370031_at	↘	↘	transporter	Cytoplasm
Others / unknown	LOC292861	kallikrein	1371080_at	↘	↘	endo-peptidase	unknown
Others / unknown	AQP6	aquaporin 6, kidney specific	1369298_at	↗	↗	transporter	Cytoplasm
others / unknown	KCNJ3	potassium inwardly-rectifying channel, subfamily J, member 3	1388292_at	↗	↗	ion channel	Plasma Membrane
Others / unknown	CTH	cystathionase	1367838_at	↗	↗	enzyme	Cytoplasm
Others / unknown	OLFR995	olfactory receptor 995	1371270_at	↘	↘	G-protein coupled receptor	Plasma Membrane
**Genes deregulated after GK11 treatment only**							
Cell signaling	CAMKK2	calcium/calmodulin-dependent protein kinase kinase 2, beta	1368753_at	-1,74		kinase	Cytoplasm
Cell signaling	RAB8B	RAB8B, member RAS oncogene family	1370539_at	1,54		enzyme	Cytoplasm
Cell signaling	PNCK	pregnancy up-regulated non-ubiquitously expressed CaM kina	1387281_a_at	↗		kinase	Cytoplasm
Cellular process - Cell Cycle	CDC2L1	cell division cycle 2 homolog (S.pombe)-like 1	1370713_at	1,53		enzyme	Cytoplasm
Cellular process - Death	KITLG	KIT ligand	1388096_at	1,58		growth factor	Extracellular Space
Cellular processes-cell survival	PRLR	prolactin receptor	1369493_at	↗		transmembrane receptor	Plasma Membrane
Cytoskeleton	SLK	STE20-like kinase (yeast)	1369037_at	-1,81		kinase	Nucleus
Cytoskeleton	RYK	RYK receptor-like tyrosine kinase	1371101_at	1,63		kinase	Plasma Membrane
Cytoskeleton	ARC	activity-regulated cytoskeleton-associated protein	1387068_at	-1,79		other	Cytoplasm
Cytoskeleton	MLLT4	myeloid/lymphoid or mixed-lineage leukemia; translocated to,	1388036_a_at	↗		other	Nucleus
Extracellular matrix	ST14	suppression of tumorigenicity 14 (colon carcinoma)	1387195_at	↗		peptidase	Plasma Membrane
Extracellular matrix	MEPE	matrix extracellular phosphoglycoprotein	1387330_at	↗		other	Extracellular Space
Extracellular matrix	NRG1	neuregulin 1	1388245_a_at	↗		growth factor	Extracellular Space
Immune response / Inflammation	GDF9	growth differentiation factor 9	1369121_at	1,66		growth factor	Extracellular Space
Immune response / Inflammation	IL13RA2	interleukin 13 receptor, alpha 2	1369266_at	1,50		transmembrane receptor	Plasma Membrane
Immune response / Inflammation	OPRK1	opioid receptor, kappa 1	1387102_at	1,76		G-protein coupled receptor	Plasma Membrane
Immune response / Inflammation	TGFA	transforming growth factor, alpha	1387450_at	↘		growth factor	Extracellular Space
Immune response / Inflammation	UCN	urocortin	1387645_at	1,57		other	Extracellular Space
Lipid Metabolism	ANXA1	annexin A1	1367614_at	1,86		other	Plasma Membrane
Lipid Metabolism	SCD2	stearoyl-Coenzyme A desaturase 2	1388253_at	-1,60		enzyme	Cytoplasm
Lipid Metabolism	INSIG1	insulin induced gene 1	1367894_at	-1,44		other	Cytoplasm
Lipid Metabolism	DGKG	diacylglycerol kinase, gamma 90kDa	1387265_at	1,75		kinase	Cytoplasm
Lipid Metabolism	CYP4A14	cytochrome P450, family 4, subfamily a, polypeptide 14	1370397_at	↗		enzyme	Cytoplasm
Response to stress / Drug	C3	complement component 3	1368000_at	1,71		peptidase	Extracellular Space
Response to stress / Drug	MX1	myxovirus (influenza virus) resistance 1, interferon-inducible p	1369202_at	1,98		enzyme	Nucleus
Response to stress / Drug	ABCC1	ATP-binding cassette, sub-family C (CFTR/MRP), member 1	1371005_at	-1,73		transporter	Plasma Membrane
Response to stress / Drug	LCN1	lipocalin 1 (tear prealbumin)	1387246_at	1,80		transporter	Extracellular Space

Family	Symbol	Entrez Gene Name	Affymetrix	Fold Chge GK11	Fold Chge MK801	Type(s) IPA classification	Location
Response to stress / Drug	MX1	myxovirus (influenza virus) resistance 1, interferon-inducible p	1387283_at	-1,68		enzyme	Nucleus
Response to stress / Drug	HSPB1	heat shock 27kDa protein 1	1367577_at	↗		other	Cytoplasm
Response to stress / Drug	ABCC6	ATP-binding cassette, sub-family C (CFTR/MRP), member 6	1368452_at	↗		transporter	Plasma Membrane
Synaptic transmission	PPP1R14A	protein phosphatase 1, regulatory (inhibitor) subunit 14A	1367813_at	-1,60		other	Cytoplasm
Synaptic transmission	CHRM3	cholinergic receptor, muscarinic 3	1369112_at	1,67		G-protein coupled receptor	Plasma Membrane
Synaptic transmission	LIN7C	lin-7 homolog C (C. elegans)	1369610_at	1,68		other	Cytoplasm
Synaptic transmission	GABRB2	gamma-aminobutyric acid (GABA) A receptor, beta 2	1369818_at	-1,56		ion channel	Plasma Membrane
Synaptic transmission	STXBP1	syntaxin binding protein 1	1370518_a_at	-2,10		transporter	Cytoplasm
Synaptic transmission	GRINL1A	glutamate receptor, ionotropic, N-methyl D-aspartate-like 1A	1371051_at	1,61		other	Nucleus
Synaptic transmission	SLC5A7	solute carrier family 5 (choline transporter), member 7	1387543_at	1,89		transporter	Plasma Membrane
Synaptic transmission	GABBR1	gamma-aminobutyric acid (GABA) B receptor, 1	1388030_a_at	1,45		G-protein coupled receptor	Plasma Membrane
Synaptic transmission	CASK	calcium/calmodulin-dependent serine protein kinase (MAGUK	1371177_a_at	↗		kinase	Plasma Membrane
Synaptic transmission	GRM8	glutamate receptor, metabotropic 8	1371242_at	↗		G-protein coupled receptor	Plasma Membrane
Transcription factor	ANKRD1	ankyrin repeat domain 1 (cardiac muscle)	1367664_at	2,21		transcription regulator	Cytoplasm
Transcription factor	SIK1	salt-inducible kinase 1	1368596_at	1,49		kinase	Cytoplasm
Transcription factor	THRB	thyroid hormone receptor, beta (erythroblastic leukemia viral (v	1387983_at	1,46		ligand-dependent nuclear receptor	Nucleus
Others / Unknown	SCAMP4	secretory carrier membrane protein 4	1367688_at	-1,48		other	unknown
Others / Unknown	PRL8A9	prolactin family8, subfamily a, member 9	1367841_a_at	2,24		other	Extracellular Space
Others / Unknown	SLC44A1	solute carrier family 44, member 1	1368480_at	-1,42		transporter	Plasma Membrane
Others / Unknown	KCNJ5	potassium inwardly-rectifying channel, subfamily J, member 5	1368560_at	1,64		ion channel	Plasma Membrane
Others / Unknown	SLC10A1	solute carrier family 10 (sodium/bile acid cotransporter family),	1368609_at	-1,41		transporter	Plasma Membrane
Others / Unknown	FUT9	fucosyltransferase 9 (alpha (1,3) fucosyltransferase)	1369258_at	-1,41		enzyme	Cytoplasm
Others / Unknown	STXBP3	syntaxin binding protein 3	1369414_at	-1,49		transporter	Plasma Membrane
Others / Unknown	VMN2R28	vomeronasal 2, receptor 28	1370664_a_at	2,55		G-protein coupled receptor	Plasma Membrane
Others / Unknown	SVS1	seminal vesicle secretory protein 1	1370860_at	1,56		enzyme	unknown
Others / Unknown	GPR88	G protein-coupled receptor 88	1387241_at	1,46		G-protein coupled receptor	Plasma Membrane
Others / Unknown	KCNK10	potassium channel, subfamily K, member 10	1369233_at	↗		ion channel	Plasma Membrane
**Genes deregulated after MK801 treatment only**							
Cell signaling	CAMK2D	calcium/calmodulin-dependent protein kinase II delta	1368834_at		-1,60	kinase	Cytoplasm
Cell signaling	RGS5	regulator of G-protein signaling 5	1369957_at		-1,41	other	Plasma Membrane
Cell signaling	PTH1R	parathyroid hormone 1 receptor	1370259_a_at		-1,66	G-protein coupled receptor	Plasma Membrane
Cell signaling	DUSP12	dual specificity phosphatase 12	1387457_at		↗	phosphatase	Nucleus
Cell signaling	AKT3	v-akt murine thymoma viral oncogene homolog 3 (protein	1387592_at		↗	kinase	Cytoplasm
Cellular processes - Adhesion	S1PR1	sphingosine-1-phosphate receptor 1	1369071_at		-1,73	G-protein coupled receptor	Plasma Membrane
Cellular processes - Adhesion	MCAM	melanoma cell adhesion molecule	1369793_a_at		1,80	other	Plasma Membrane
Cellular processes - Adhesion	ST8SIA3	ST8 alpha-N-acetyl-neuraminide alpha-2,8-sialyltransferase 3	1387435_at		-1,57	enzyme	Cytoplasm
Cellular processes - Growth	LSAMP	limbic system-associated membrane protein	1370550_at		-1,45	other	Plasma Membrane

Family	Symbol	Entrez Gene Name	Affymetrix	Fold Chge GK11	Fold Chge MK801	Type(s) IPA classification	Location
Cellular processes - Differentiation	PROM1	prominin 1	1368008_at		-1,70	other	Plasma Membrane
Cellular processes - Differentiation	CSRP2	cysteine and glycine-rich protein 2	1370282_at		-1,55	other	Nucleus
Cellular processes - Migration	PDE1C	phosphodiesterase 1C, calmodulin-dependent 70kDa	1371219_a_at		1,82	enzyme	Cytoplasm
Cellular processes-migration	NTN1	netrin 1	1398269_at		↗	other	Extracellular Space
Cellular processes - Proliferation	GOLPH3	golgi phosphoprotein 3 (coat-protein)	1369571_at		1,76	other	Cytoplasm
Cellular processes - Proliferation	TRPC6	transient receptor potential cation channel, subfamily C, memb	1370139_a_at		-1,76	ion channel	Plasma Membrane
Cellular processes - Proliferation	GHRHR	growth hormone releasing hormone receptor	1370730_a_at		1,73	G-protein coupled receptor	Plasma Membrane
Cellular processes - Proliferation	CCND1	cyclin D1	1371150_at		↘	other	Nucleus
Cellular processes - Survival	AURKAIP1	aurora kinase A interacting protein 1	1398916_at		-1,60	enzyme	Nucleus
Cellular processes - Death	SCN3A	sodium channel, voltage-gated, type III, alpha subunit	1369691_at		-1,54	ion channel	Plasma Membrane
Cellular processes -Death	DDIT4	DNA-damage-inducible transcript 4	1368025_at		1,72	other	Cytoplasm
Cellular processes -Death	ADCY8	adenylate cyclase 8 (brain)	1387179_at		-1,61	enzyme	Plasma Membrane
Cellular processes -Death	AXIN2	axin 2	1387184_at		-1,53	other	Cytoplasm
Cytoskeleton	EMD	emerin	1370017_at		1,66	other	Nucleus
Cytoskeleton	EPB41L3	erythrocyte membrane protein band 4.1-like 3	1370503_s_at		-1,60	other	Plasma Membrane
Cytoskeleton	MAP6	microtubule-associated protein 6	1371087_a_at		-1,52	other	Cytoplasm
Cytoskeleton	BAIAP2	BAI1-associated protein 2	1374117_at		2,04	kinase	Plasma Membrane
Cytoskeleton	ACTG2	actin, gamma 2, smooth muscle, enteric	1386869_at		↗	other	Cytoplasm
Cytoskeleton	MYOC	myocilin, trabecular meshwork inducible glucocorticoid respon	1387313_at		↗	other	Cytoplasm
Extracellular matrix	CTGF	connective tissue growth factor	1367631_at		2,13	growth factor	Extracellular Space
Extracellular matrix	MMP24	matrix metallopeptidase 24 (membrane-inserted)	1368595_at		1,49	peptidase	Extracellular Space
Extracellular matrix	FN1	fibronectin 1	1370234_at		1,51	enzyme	Plasma Membrane
Extracellular matrix	VCAN	versican	1388054_a_at		-2,15	other	Extracellular Space
Extracellular matrix	EPHA7	EPH receptor A7	1388061_a_at		-1,90	kinase	Plasma Membrane
Extracellular matrix	LECT1	leukocyte cell derived chemotaxin 1	1387164_at		↗	other	Extracellular Space
Immune response / Inflammation	PLAT	plasminogen activator, tissue	1367800_at		2,20	peptidase	Extracellular Space
Immune response / Inflammation	SGK1	serum/glucocorticoid regulated kinase 1	1367802_at		2,13	kinase	Cytoplasm
Immune response / Inflammation	TGFB3	transforming growth factor, beta 3	1367859_at		1,55	growth factor	Extracellular Space
Immune response / Inflammation	SCG2	secretogranin II (chromogranin C)	1368044_at		1,86	cytokine	Extracellular Space
Immune response / Inflammation	ADAMTS1	ADAM metallopeptidase with thrombospondin type 1 motif, 1	1368223_at		2,12	peptidase	Extracellular Space
Immune response / Inflammation	PTGS2/ Cox2	prostaglandin-endoperoxide synthase 2 (prostaglandin G/	1368527_at		**1,62**	enzyme	Cytoplasm
Immune response / Inflammation	ACVRL1	activin A receptor type II-like 1	1368553_at		1,81	kinase	Plasma Membrane
Immune response / Inflammation	P2RY12	purinergic receptor P2Y, G-protein coupled, 12	1368728_at		-2,05	G-protein coupled receptor	Plasma Membrane
Immune response / Inflammation	IL18BP	interleukin 18 binding protein	1369031_at		1,64	other	Extracellular Space
Immune response / Inflammation	CD47	CD47 molecule	1369559_a_at		-1,52	other	Plasma Membrane
Immune response / Inflammation	SLC16A7	solute carrier family 16, member 7 (monocarboxylic acid trans	1370609_a_at		-1,62	transporter	Plasma Membrane

Family	Symbol	Entrez Gene Name	Affymetrix	Fold Chge GK11	Fold Chge MK801	Type(s) IPA classification	Location
Immune response / Inflammation	SPON2	spondin 2, extracellular matrix protein	1370847_at		1,96	other	Extracellular Space
Immune response / Inflammation	SLC2A1	solute carrier family 2 (facilitated glucose transporter), membe	1370848_at		1,61	transporter	Plasma Membrane
Immune response / Inflammation	NOS3	nitric oxide synthase 3 (endothelial cell)	1371166_at		**1,89**	enzyme	Cytoplasm
Immune response / Inflammation	GM5068	predicted gene 5068	1375170_at		1,82	other	unknown
Immune response / Inflammation	IL6R	interleukin 6 receptor	1386987_at		**1,82**	transmembrane receptor	Plasma Membrane
Immune response / Inflammation	RGS2	regulator of G-protein signaling 2, 24kDa	1387074_at		1,58	other	Nucleus
Immune response / Inflammation	NPR2	natriuretic peptide receptor B/guanylate cyclase B (atrionatriur	1387099_at		1,70	G-protein coupled receptor	Plasma Membrane
Immune response / Inflammation	IGFBP6	insulin-like growth factor binding protein 6	1387625_at		-1,64	other	Extracellular Space
Immune response / Inflammation	IFI27	interferon, alpha-inducible protein 27	1387770_at		-1,67	other	Cytoplasm
Immune response / Inflammation	SLC9A3R2	solute carrier family 9 (sodium/hydrogen exchanger), member	1387976_at		1,86	transporter	Plasma Membrane
Immune response / Inflammation	TINAGL1	tubulointerstitial nephritis antigen-like 1	1367650_at		↗	transporter	Extracellular Space
Immune response / Inflammation	LTA	lymphotoxin alpha (TNF superfamily, member 1)	1368722_at		↗	cytokine	Extracellular Space
Immune response / Inflammation	ITGB7	integrin, beta 7	1371106_at		↗	transmembrane receptor	Plasma Membrane
Immune response / Inflammation	PLA2G10	phospholipase A2, group X	1387250_at		↗	enzyme	Cytoplasm
Insuline signaling	RLN1	relaxin 1	1368495_at		1,60	other	Extracellular Space
Insuline signaling	IRS3	insulin receptor substrate 3	1368486_at		↗	other	Nucleus
Lipids metabolism	ACSL6	acyl-CoA synthetase long-chain family member 6	1368182_at		-1,52	enzyme	Cytoplasm
Lipids metabolism	ASPA	aspartoacylase (Canavan disease)	1368563_at		1,75	enzyme	unknown
Lipids metabolism	PAFAH1B2	platelet-activating factor acetylhydrolase 1b, catalytic subunit 2	1369641_at		1,45	enzyme	Cytoplasm
Lipids metabolism	PEX5L	peroxisomal biogenesis factor 5-like	1370833_at		-1,49	other	Cytoplasm
Lipids metabolism	PPAP2B	phosphatidic acid phosphatase type 2B	1370950_at		-1,61	phosphatase	Plasma Membrane
Lipids metabolism	ACOX2	acyl-Coenzyme A oxidase 2, branched chain	1371137_at		1,70	enzyme	Cytoplasm
Lipids metabolism	SC5DL	sterol-C5-desaturase (ERG3 delta-5-desaturase homolog, S. c	1387926_at		1,76	enzyme	Cytoplasm
Lipids metabolism	ACACA	acetyl-Coenzyme A carboxylase alpha	1387538_at		↗	enzyme	Cytoplasm
Response to stress / drug	DUSP1	dual specificity phosphatase 1	1368146_at		3,65	phosphatase	Nucleus
Response to stress / drug	RTN3	reticulon 3	1368807_at		1,66	other	Cytoplasm
Response to stress / drug	KCNJ12	potassium inwardly-rectifying channel, subfamily J, member 12	1369795_at		-1,66	ion channel	Plasma Membrane
Response to stress / drug	STAT3	signal transducer and activator of transcription 3 (acute-phase	1370224_at		1,78	transcription regulator	Nucleus
Response to stress / drug	CA2	carbonic anhydrase II	1386922_at		-1,47	enzyme	Cytoplasm
Response to stress / drug	JUNB	jun B proto-oncogene	1387788_at		1,76	transcription regulator	Nucleus
Response to stress / drug	HYOU1	hypoxia up-regulated 1	1370665_at		↗	other	Cytoplasm
Response to stress / drug	CYP2C9	cytochrome P450, family 2, subfamily C, polypeptide 9	1387328_at		↗	enzyme	Cytoplasm
Synapse remodelling	WIF1	WNT inhibitory factor 1	1369203_at		1,70	other	Extracellular Space
Synaptic transmission	NSF	N-ethylmaleimide-sensitive factor	1369690_at		-1,56	transporter	Cytoplasm
Synaptic transmission	CORT	cortistatin	1387337_at		1,58	other	Extracellular Space
Synaptic transmission	GRM3	glutamate receptor, metabotropic 3	1388189_at		-1,52	G-protein coupled receptor	Plasma Membrane
Transcription factor	CITED2	Cbp/p300-interacting transactivator, with Glu/Asp-rich carboxy	1367602_at		2,28	transcription regulator	Nucleus

Family	Symbol	Entrez Gene Name	Affymetrix	Fold Chge GK11	Fold Chge MK801	Type(s) IPA classification	Location
Transcription factor	TSC22D3	TSC22 domain family, member 3	1367771_at		2,34	transcription regulator	Nucleus
Transcription factor	EGR1	early growth response 1	1368321_at		-2,32	transcription regulator	Nucleus
Transcription factor	USF2	upstream transcription factor 2, c-fos interacting	1368591_at		-1,81	transcription regulator	Nucleus
Transcription factor	ID2	inhibitor of DNA binding 2, dominant negative helix-loop-helix protein	1368870_at		-1,52	transcription regulator	Nucleus
Transcription factor	NR4A2	nuclear receptor subfamily 4, group A, member 2	1369007_at		2,25	ligand-dependent nuclear receptor	Nucleus
Transcription factor	FHL2	four and a half LIM domains 2	1369313_at		-1,85	other	Nucleus
Transcription factor	HOXA1	homeobox A1	1369544_a_at		2,09	transcription regulator	Nucleus
Transcription factor	PPARGC1A	peroxisome proliferator-activated receptor gamma, coactivator	1370089_at		-2,10	transcription regulator	Nucleus
Transcription factor	ZNF597	zinc finger protein 597	1370705_at		1,56	other	Nucleus
Transcription factor	FHL2	four and a half LIM domains 2	1371951_at		-1,73	other	Nucleus
Transcription factor	NR4A1	nuclear receptor subfamily 4, group A, member 1	1386935_at		1,43	ligand-dependent nuclear receptor	Nucleus
Transcription factor	ID1	inhibitor of DNA binding 1, dominant negative helix-loop-helix	1387028_a_at		1,47	transcription regulator	Nucleus
Transcription factor	CEBPB	CCAAT/enhancer binding protein (C/EBP), beta	1387087_at		3,86	transcription regulator	Nucleus
Transcription factor	ZRANB2	zinc finger, RAN-binding domain containing 2	1387117_at		-1,65	transcription regulator	Nucleus
Transcription factor	PHOX2A	paired-like homeobox 2a	1387238_at		2,10	transcription regulator	Nucleus
Transcription factor	EGR2	early growth response 2	1387306_a_at		-1,69	transcription regulator	Nucleus
Transcription factor	CEBPD	CCAAT/enhancer binding protein (C/EBP), delta	1387343_at		1,64	transcription regulator	Nucleus
Transcription factor	EGR4	early growth response 4	1387442_at		1,57	transcription regulator	Nucleus
Transcription factor	FST	follistatin	1387843_at		-1,69	other	Extracellular Space
Transcription factor	ZFP36	zinc finger protein 36, C3H type, homolog (mouse)	1387870_at		2,08	transcription regulator	Nucleus
Transcription factor	MAFB	v-maf musculoaponeurotic fibrosarcoma oncogene homolog B	1387947_at		1,63	other	Nucleus
Transcription factor	PRPF18	PRP18 pre-mRNA processing factor 18 homolog (S. cerevisia	1388005_at		1,92	transporter	Nucleus
Transcription factor	NR4A3	nuclear receptor subfamily 4, group A, member 3	1369217_at		↗	ligand-dependent nuclear receptor	Nucleus
Transcription factor	KLF4	Kruppel-like factor 4 (gut)	1387260_at		↗	transcription regulator	Nucleus
Transcription factor	FOSL2	FOS-like antigen 2	1387530_a_at		↗	transcription regulator	Nucleus
Other / Unknown	IFRD1	interferon-related developmental regulator 1	1367795_at		2,00	other	Nucleus
Other / Unknown	CP	ceruloplasmin (ferroxidase)	1368420_at		1,56	enzyme	Extracellular Space
Other / Unknown	KPNB1	karyopherin (importin) beta 1	1368573_at		-1,81	transporter	Nucleus
Other / Unknown	CYP1B1	cytochrome P450, family 1, subfamily B, polypeptide 1	1368990_at		1,61	enzyme	Cytoplasm
Other / Unknown	SLC5A5	solute carrier family 5 (sodium iodide symporter), member 5	1369020_at		1,63	transporter	Plasma Membrane
Other / Unknown	SC65	synaptonemal complex protein SC65	1369805_at		1,78	other	Nucleus
Other / Unknown	PORF1	preoptic regulatory factor 1	1369885_at		2,05	other	Extracellular Space
Other / Unknown	C5ORF62	chromosome 5 open reading frame 62	1370408_at		1,43	ion channel	unknown
Other / Unknown	SCAMP1	secretory carrier membrane protein 1	1370978_at		-1,57	transporter	Cytoplasm
Other / Unknown	SCAPER	S-phase cyclin A-associated protein in the ER	1371093_at		-1,46	other	unknown
Other / Unknown	SLC14A2	solute carrier family 14 (urea transporter), member 2	1371205_at		1,66	transporter	Plasma Membrane
Other / Unknown	PC	pyruvate carboxylase	1386917_at		1,94	enzyme	Cytoplasm

Family	Symbol	Entrez Gene Name	Affymetrix	Fold Chge GK11	Fold Chge MK801	Type(s) IPA classification	Location
Other / Unknown	GUCY1A3	guanylate cyclase 1, soluble, alpha 3	1387079_at		-1,64	enzyme	Cytoplasm
Other / Unknown	CBS	cystathionine-beta-synthase	1387178_a_at		1,73	enzyme	Cytoplasm
Other / Unknown	KCNV1	potassium channel, subfamily V, member 1	1387881_at		-1,84	ion channel	unknown
Other / Unknown	SLC22A24	solute carrier family 22, member 24	1388172_at		1,86	transporter	unknown
Other / Unknown	VPS52	vacuolar protein sorting 52 homolog (S. cerevisiae)	1369668_x_at		↗	other	Cytoplasm
Other / Unknown	HTR5B	5-hydroxytryptamine (serotonin) receptor 5B	1388219_at		↗	G-protein coupled receptor	Plasma Membrane
